# Supplementary material for: Two distinct host-specialized fungal species cause white-nose disease in bats
Source: Nature. 2025 May 28;642(8069):1034–40. doi: 10.1038/s41586-025-09060-5 (PMC12222008; doi:10.1038/s41586-025-09060-5)
Supplement: Supplementary file 1 — This file includes Supplementary Notes 1 and 2, Supplementary Figs. 1 and 3–6 and Supplementary Tables 6–18 that support the main article with raw data and summaries. Supplementary references are also included. [file 41586_2025_9060_MOESM1_ESM.pdf]

nature portfolio

https://doi.org/10.1038/s41586-025-09060-5

Supplementary information

Two distinct host-specialized fungal species cause white-nose disease in bats

In the format provided by the authors and unedited

Supporting Information for

Two distinct host-specialized fungal species cause white-nose disease in bats

Nicola M. Fischer, Imogen Dumville, Benoit Nabholz, Violeta Zhelyazkova, Ruth-Marie Stecker, Anna S. Blomberg, Serena E. Dool, Marcus Fritze, Marie-Ka Tilak, Andriy-Taras Bashta, Clothilde Chenal, Anna-Sophie Fiston-Lavier, Sebastien J. Puechmaille

This PDF file includes:

Supplementary Text S1 to S2
Figures S1 and S3 to S6
Tables S6 to S18
SI References

Other supporting materials for this manuscript include the following:

Tables S1-S5 (.xlsx)
Figure S2 (.pdf)

Supplementary Text S1 to S2

Supplementary Text S1. Further insights regarding the potential mechanisms that underlie the initial introduction of *P. destructans* (*Pd*-1) to North America.

Identifying the origin of clonal *Pd*-1 isolates in North America is a critical first step in understanding *P. destructans* invasion and developing strategies to prevent future introductions of *Pd*-1 or *Pd*-2 into new geographic regions. Herein, we present strong evidence that Podillia, a region in Ukraine, is the region of origin of *Pd*-1's introduction to North America. While this discovery significantly advances our understanding of white-nose disease, pinpointing the precise mechanism of introduction remains a complex challenge. As with many invasive species or pathogen introductions—including those affecting humans and domestic animals—definitive proof is nearly impossible to obtain. However, multiple independent lines of evidence provide a robust and consistent explanation for *Pd*'s transcontinental spread.

1. High-risk cave environment

Firstly, Podillia is home to some of the largest cave systems and karst landscapes in Europe, including Optymistychna cave, the continent's longest, spanning over 267 km. These extensive maze caves attract numerous recreational cavers, researchers and speleotourists from around the world. Notably, two of our co-authors have visited some of these caves. Their intricate networks of corridors often require cavers to crawl through narrow passages, increasing contact between clothing, gear, and the cave environment—where viable *Pd* is known to persist[1-3].

2. Persistence on caving gear

Secondly, *Pd* is sensitive to UV light, including natural daylight, which limits its survival under prolonged exposure[4]. However, caving gear provides an ideal sheltered environment for the fungus, as it is used primarily in caves and typically stored in bags during travel. This makes it an effective vector for transporting spores between caves and across regions. Direct detection of *Pd* on gear used in *Pd*-positive sites confirms its ability to persist and spread via caving equipment[5]. Additionally, laboratory studies show that *Pd* spores can remain viable on gear and clothing for several weeks at room temperature, further enabling their transcontinental transport[5].

3. Documented travel pathways

Thirdly, we examined available data on caver and researcher travel between cave systems in Podillia and North America before 2006. Following the collapse of the USSR in 1991, previously restricted caves in Eastern Europe, including Ukraine, became accessible to international cavers, particularly from the United States[6]. The late 1990s to early 2000s—aligning with the estimated introduction of *Pd* to North America—saw a surge in international caving expeditions to and from Podillia. We confirmed multiple caving trips between Podillia and North America during this period[7-9], including to the Albany/Schoharie region of New York State (e.g.[10,11]), where *Pd* was detected before 2006[12].Access to caves is not too strictly regulated in Ukraine, thus the actual number of caving trips to Podillia can only be higher than the ones documented. The convergence of genetic, historical, ecological, and experimental evidence provides strong support that caving activities—specifically through contaminated gear—were the primary mechanism of *Pd*'s transport and introduction to North America.

4. Exclusion of bat-mediated transmission:

Finally, the geographic origin of *Pd*-1 further supports human-mediated introduction. The source population is located in the heart of the European continent (Ukraine), and there are no bat species shared between Eurasia and North America. This absence of natural bat-mediated dispersal routes makes a direct zoonotic transmission across continents highly improbable. Instead, the introduction of *Pd* to North America aligns with other documented cases of anthropogenic pathogen spread, such as the human-facilitated movement of *Pd* between Eastern and Western North America[13].

5. Conservation and Management Implications

The implications of this mode of transport for conservation are significant. Further introductions of *Pd*-1 or *Pd*-2 outside their natural range could exacerbate population declines in bat species. Given previous introductions (e.g., Europe to North America, and between Eastern and Western North America;[13]), and a previous risk assessment conducted in Australia, which concluded that in the next 10 years, the introduction of *Pd* is very likely to almost certain, future long-distance introductions are highly probable.

An essential management strategy to mitigate further spread is rigorous decontamination of caving gear. Experimental evidence shows that washing caving equipment reduces viable *Pd* spores by 94% (hand washing) to 95% (machine washing at 30°C), with even greater efficacy at higher temperatures and with detergent[5]. These results demonstrate that simple, cost-effective measures can significantly reduce transmission risk. Immediate implementation of decontamination guidelines is crucial. Raising awareness among scientists, recreational cavers, and tour organisers is essential to prevent additional introductions of *Pd* to unaffected regions[14].

Supplementary Text S2. List of contributors who helped with sample collection in the field:

A. Bezard, A. Kubátová, Aleksandra Lange, Alessandra Peron, Alex Lefevre, Alexander Lazarov, Alexandra Telea, Alexandre Cartier, Alina Larion, Alphonse Malpel, Amanda Davies, Andres Beck, Andrew Brinckman, Andriy Melnychuk, Andrzej Kepel, Andrzej Wojtaszewski, Angel Ivanov, Àngel Torrent, Ann Lenaerts, Anna Roswag, Anna Suvorova, Anne-Jilke Haarsma, Anne Petzold, Annika Breitfelder, Anthony Lane, Anthony Le Nozahic, Anthony Nickson, Antonia Hubancheva, Artem Tarasov, Ash Murray, Atanas Stavrev, Axel Donning, Axel Griesau, Axel Keusemann, Bart Mulkens, Benjamin Meme-Lafond, Bernd Ohlendorf, Bernhard Walk, Beytullah Özkan, Blanka Lehotská, Boris Petrov, Brian Briggs, Brigitte Meiswinkel, Carlos Ibáñez, Carsten Dense, Catherine Reilly, Chris Vine, Christian Dietz, Christian Jungmann, Christian Sebening, Christoph Treß, Christophe Borel, Christophe Parisot, Christopher Paton, Claudi Gebhart, Clemens Kliesch, Colin Morris, Corentin Le Floch, Csaba Jére, Damian Celiński, Dana Wagemakers, Daniel Eva, Daniela Hamidovic, Daniela Pilgrim, Daniela Schmieder, Daniela Wieser, Dave Hughes, David Anderson, David Aupermann, David Dodds, David Endacott, David García Jiménez, David Hellmann, David Patterson, David Wills, Didier Montfort, Dieter Hülshoff, Dieter Sulzbacher, Dimitar Kunev, Dirk Karoske, Dragoş Bălăşoiu, Ebbe Nytors, Eeva-Maria Kyheröinen, Egoitz Salsamendi, Elena Migens Maqueda, Emrah Çoraman, Erich Taube, Ernst Auer, Eva Kriner, Ewa Przepiorka, Fabio Bontadina, Fabio Suppini, Fiona Parker, Florian Gloza-Rausch, Francesco Grazioli, Frank Meisel, Frauke Meier, Frédéric Forget, Frédéric Touzalin, Fulgencio Lison, Gabriella Krivek, Gaël Verat, Gary Shears, Georg Warnke, Gerald Kerth, Gerald Larcher, Giazarian, Goran Rnjak, Gregory Beneux, Grzegorz Apoznański, Grzegorz Lesinski, Guilia Console, Gunars Petersons, Gunther Capo, Gustav Dinger, Gwenaelle Hurpy, Gwendoline Dumenil, H. Bandouchova, H. Seimers, Hannes Köble, Harald Mixanig, Heino Hauf, Helen Miller, Henryk Hörner, Holger Schütt, Hubert Baltus, Iain Hysom, Ian Bond, Ilaria Vaccarelli, Ilona Imoberdorf, Ilze Brila, Inazio Garin, Ingrid Heißen, Ingrid Oftedal, Irbin Manuel Veliz Isidro, Ireneusz Ruczynski, Irina Pocora, Irina Würtele, István Csősz, Ivailo Borissov, Ivan Napotnik, Ivana Budinski, J. Flousek, J. Nogueras, J. Pikula, J. Zukal, J.L. Gathoye, Jamie Shadbolt, Jan Boshmer, Jane Harris, Jane Sedgeley-Strachan, Jasmin Pašić, Jasminko Mulaomerović, Jean-Yves Courtois, Jean Guhring, Jenny Harris, Jens Berg, Jens Krüger, Jens Rydell, Jeroen van der Kooij, Joachim Frömert, John Haddow, Johnny de Jong, Jörn Horn, Jose Siles, Juan R. Boyero, Julia Prüger, Juliane Schatz, Jurgis Suba, Justyna Błesznowska, Karina Jungmann, Karsten Passior, Katharina Bürger, Kathy Warden, Kees Mostert, Kerstin Genz, Klaus Heck, Kristof De Clercq, Krum Sirakov, Krzysztof Piksa, Kseniia Kravchenko, Laura Torrent, Laurence Florian, Laurent Arthur, Lauri Lutsar, Lea Bütje, Lena Godlevska, Lena Grosche, Lide Jimenez, Lilian Girard, Lionel L'Hoste, Lisa Worledge, Llorenc Capella Ripoll, Loic Robert, Lotte Gielis, Lucretia Deplazes, Ludovic Jouve, Luis Vicente, Luisa Rodrigues, Lyn Wells, M. Kubešová, M. Orlova, Magda Milczarska, Maik Korreng, Manfred Keller, Manuel Graf, Manuela Schult, Mara Calvini, Marcin Rusinski, Maria Das Neves Paiva Cardoso, Marion Laprun, Markus Melber, Markus Milchram, Markus Schmidberger, Markus Thies, Martin Biedermann, Martin Harder, Martin Koch, Martin Starrach, Martina Palmer, Mathijs Borms, Matija Perne, Matthias Göttsche, Matthias Hammer, Matthias Weiß, Matthias Zizelmann, Mauro Mucedda, Mechthild Höller, Michael Frede, Miguel Àngel Fuentes Rosua, Mike Debret, Mirna Mazija, Momchil Naydenov, Monika Podgorelec, Morten Elmeros, N. Martinkova, Nataša Sivec, Nia Toshkova, Nick Tribe, Nicolas Cayssiols, Nicolas Fasel, Nicola Fischer, Niklois Jungbluth, Nina Hagner-Wahlsten, Norbert Röse, Nuno Pinto, O. Orlov, Oliver Kalda, Oleksandr Vikyrchak, Olvido Tejedor, Oscar de Paz, P. Blažková, P. Schnitzerová, P. Táje, P. Tájek, Paola Culasso, Pascal Bellion, Pascal Giosa, Pascal Verdeyroux, Patty Briggs, Paul Hope, Paweł Kmiecik, Per IngeVærnesbranden, Peter Busse, Peter Heubes, Peter Holtz, Peter Smith, Petra Gatz, Petra Žvorc, Petro Ploshchanskyi, Philippe Defernez, Philippe Theou, Pierre-Emmanuel Bastien, Piotr Zielinski, Primož Presetnik, Quentin Smits, Radek Lučan, Radostina Tsoneva, Rainer Marcek, Ralf Hansen, Ralf Koch, Rasit Bilgin, Rauno Kalda, Reimund Francke, Reinhard Koch, Rémi Hanotel, Rich Flight, Roberto Toffoli, Robin Moffitt, Ruddy Cors, S. Rebrov, Sabine Lind, Sabine Portig, Sam Dyer, Sándor Boldogh, Sandra Möller, Sebastian Petters, Sebastien Puechmaille, Serbülent Pakzuz, Serena Dool, Serena Magagnoli, Sheelagh Kerry, Shirley Thompson, Simone Pysarczuk, Stamen Dimitrov, Stanimira Deleva, Stefan Schürmann, Steffi Pfeiffer, Stephanie Wohlfahrt, Steve Parker, Stoyan Goranov, Sue Lane, Susan Kerwin, Susanne Rosenau, Szilárd Bücs, T. Juhnke, Tamás Görföl, Tarik Dervović, Tea Knapič, Teodor Jhotev, Thomas Bormann, Thomas Cheyrezy, Thomas Frank, Thomas Kuß, Thomas Le Campion, Thomas Lilley, Tiago Brito, Tina Aughney, Tino Staudt, Todor Karakiev,

Tom Hastings, Tom McOwat, Tomasz Kokurewicz, Toni Watt, Tony Lane, Torsten Blohm, Tsvetan Ostromsky, Ulrich Zöphel, V. Kovacova, V. Lensinger, V.S. Crukov, Vesselin Zhelyazkov, Victor Senderov, Victoria Nistreanu, Viktor Ilyukha, Viorel Pocora, Vita Hommersen, Vitaliy Guckov, Vivien Sottejean, Vladislav Caldari, Vlashenko, Volker Kubisch, Weigert Steen, Wigbert Schorcht, Winfried Krämer, Wolfgang Fiedler, Wolfgang Otremba, Wolfgang Rackow, Wolfgang Strittmatter, Xavier Mestdagh, Yana Dimova, Yann Gager, Yann Le Bris, Yannick Beucher, Yvon Guenescheau, Zuzanna Halat.

Supplementary Figures S1 and S3 to S6

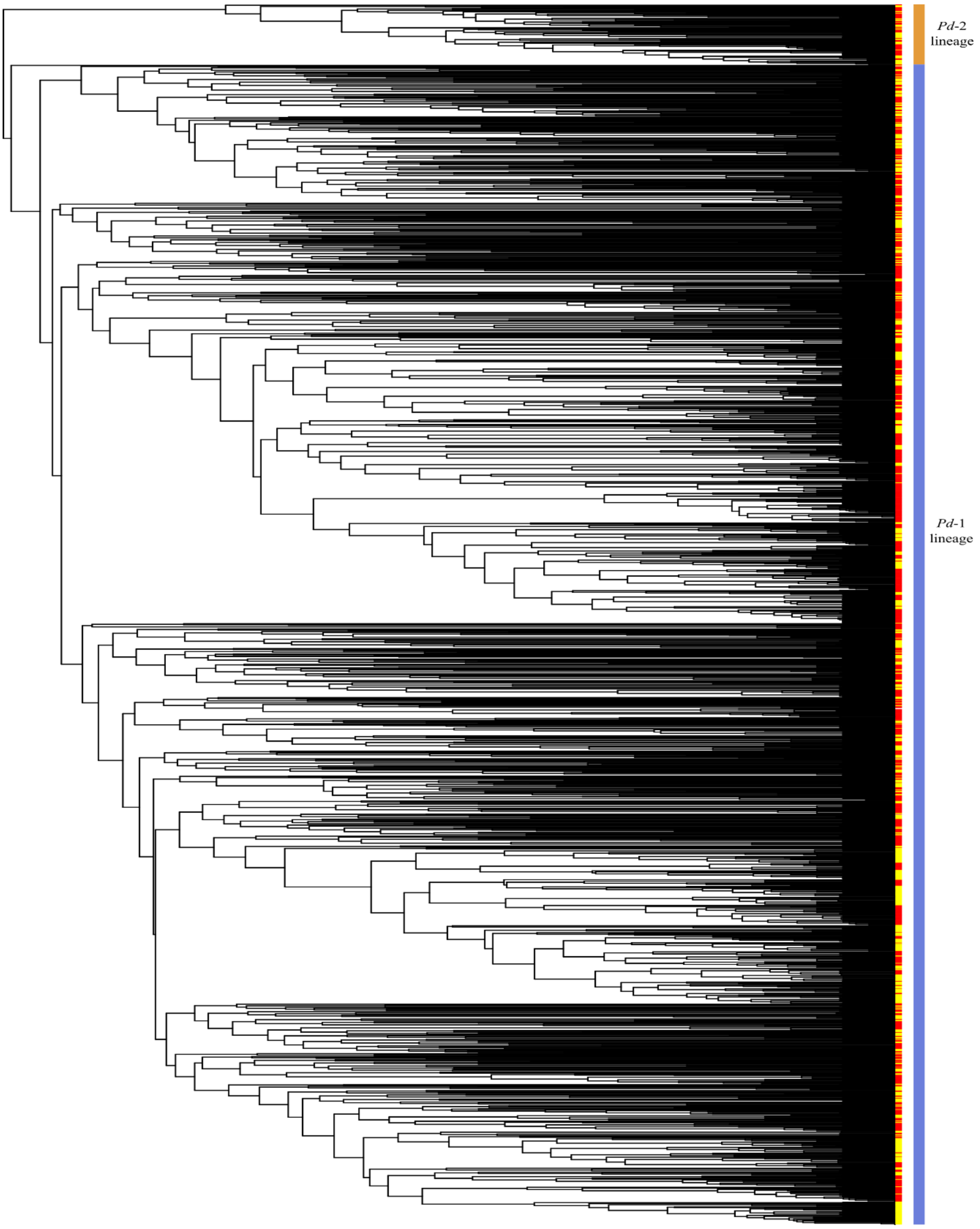

Fig. S1. Phylogenetic tree representing the relationship between the 1,866 multi-locus genotypes obtained from genotyping at 18 microsatellite loci a set of 5,479 *P. destructans* isolates. The two identified monophyletic clades (*Pd*-1 and *Pd*-2) are depicted by the blue and orange vertical bars to the right. The tree is constructed via the UPGMA algorithm applied to the Nei's "Da" genetic distance[15]. Next to the tips, the mating types MAT 1-1 and MAT 1-2 are depicted by red and yellow horizontal bars respectively. For legibility issues, individual labels of the isolates are not presented (see Figure S2 for labels).

Fig. S2 - (Provided as separate pdf file)
Phylogenetic tree depicting the relationship between the 5,479 *P. destructans* isolates based on their genotype at 18 microsatellite loci. Next to the tip, the colour of the square represents (from left to right) the clade (*Pd*-1, *Pd*-2), the bat species from which the isolate was sampled (if the sample was collected from the environment, it is left blank), and the country of origin. The tip labels consist of the isolate identifier (e.g., Gd_00293-aad), the substrate from which the isolate was sampled (if sampled from a bat, abbreviations of bat species as in the legend of Table S1), the abbreviated names of the countries, and lastly, the site number as presented in Table S1. All of this metadata is also available in Table S1.

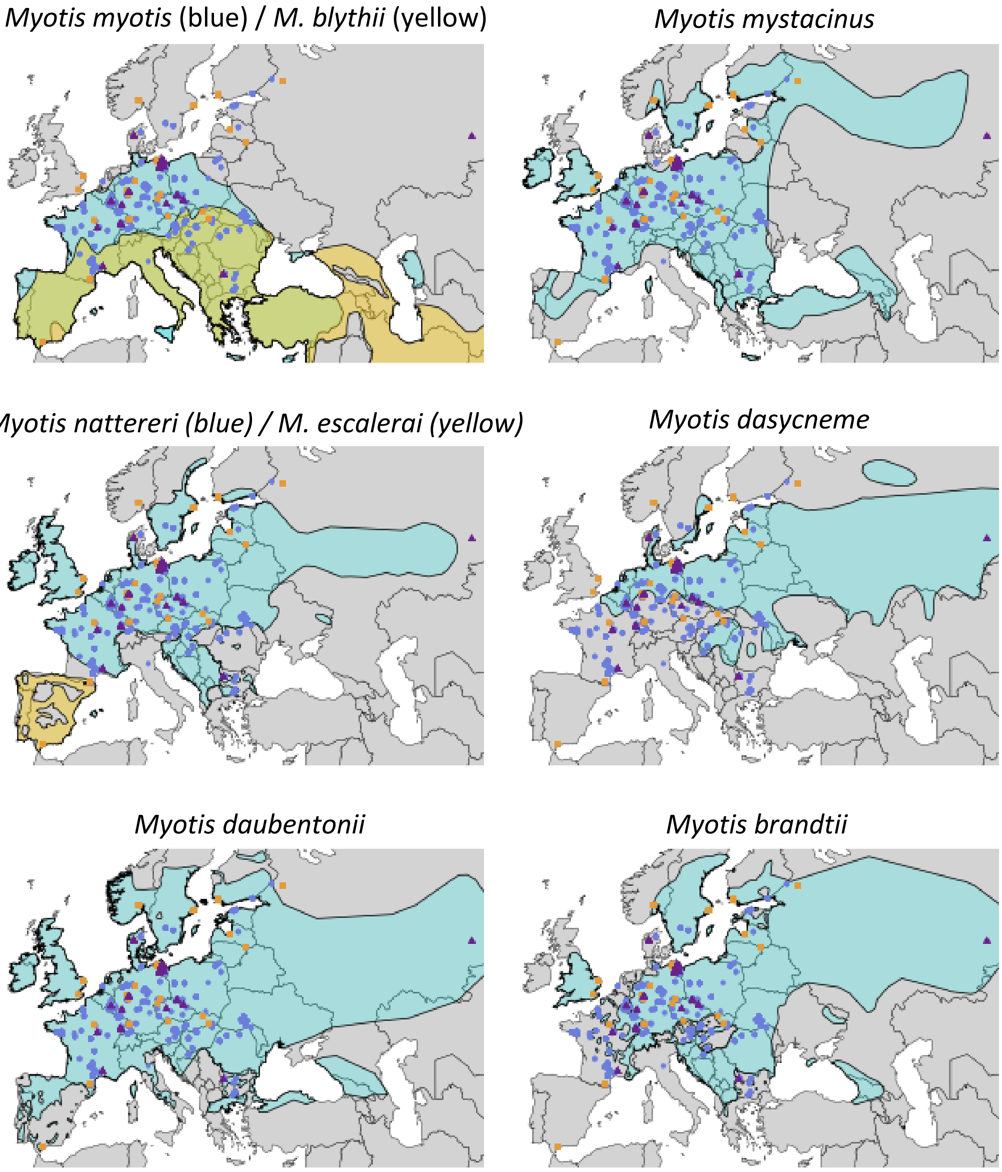

Fig. S3. Distribution of the top six most commonly sampled bat species/species combinations. Distribution data obtained from IUCN Red List of Threatened Species™[16] (https://www.iucnredlist.org) as blue/yellow shaded areas on individual maps with sampling locations of *Pd*-1 (blue), *Pd*-2 (orange) and both clades (purple) dots overlaid. Due to recent taxonomic change in the *Myotis nattereri* species complex, *M. crypticus* and *M. nustrale* maps are not available from the IUCN and the *Myotis nattereri s.s.* is missing some areas of presence. *M. nattereri s.s.* is present across Romania and *M. crypticus* is present across the Italian Peninsula, Southern France and Northern Iberia while *M. nustrale* is present in Corsica (reviewed in [17]).

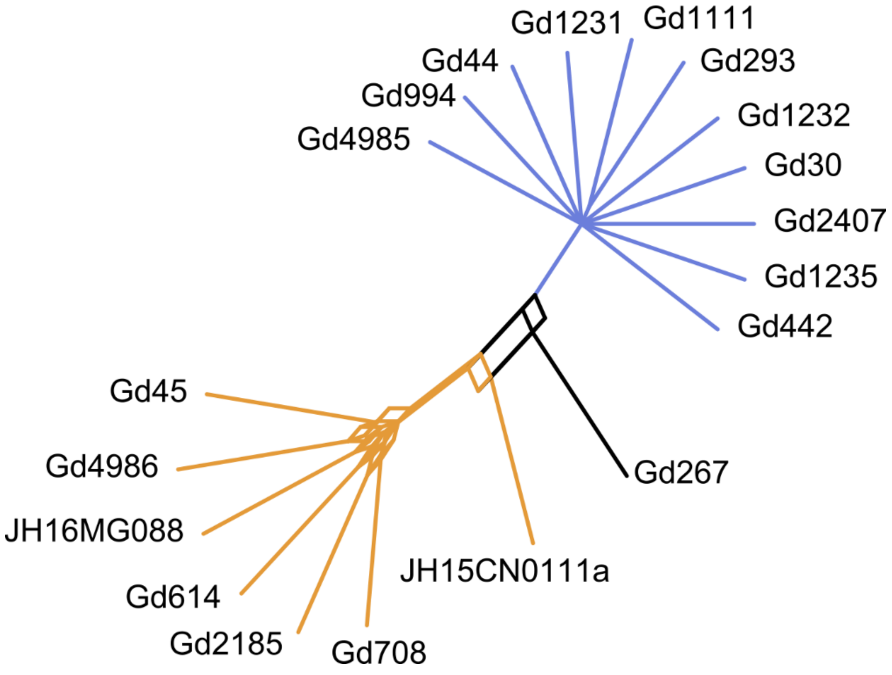

Fig. S4. Consensus network depicting high congruence between the 664 BUSCO gene trees (same dataset as in Figure 2b). The network was produced with a proportion of 0.10, where a split must be present in 10% of trees in order to be represented in the network. *Pd*-1 is represented in blue, *Pd*-2 in orange, and the outgroup (Gd267; *Pseudogymnoascus sp*.) in black. See Table S10 for information on the samples.

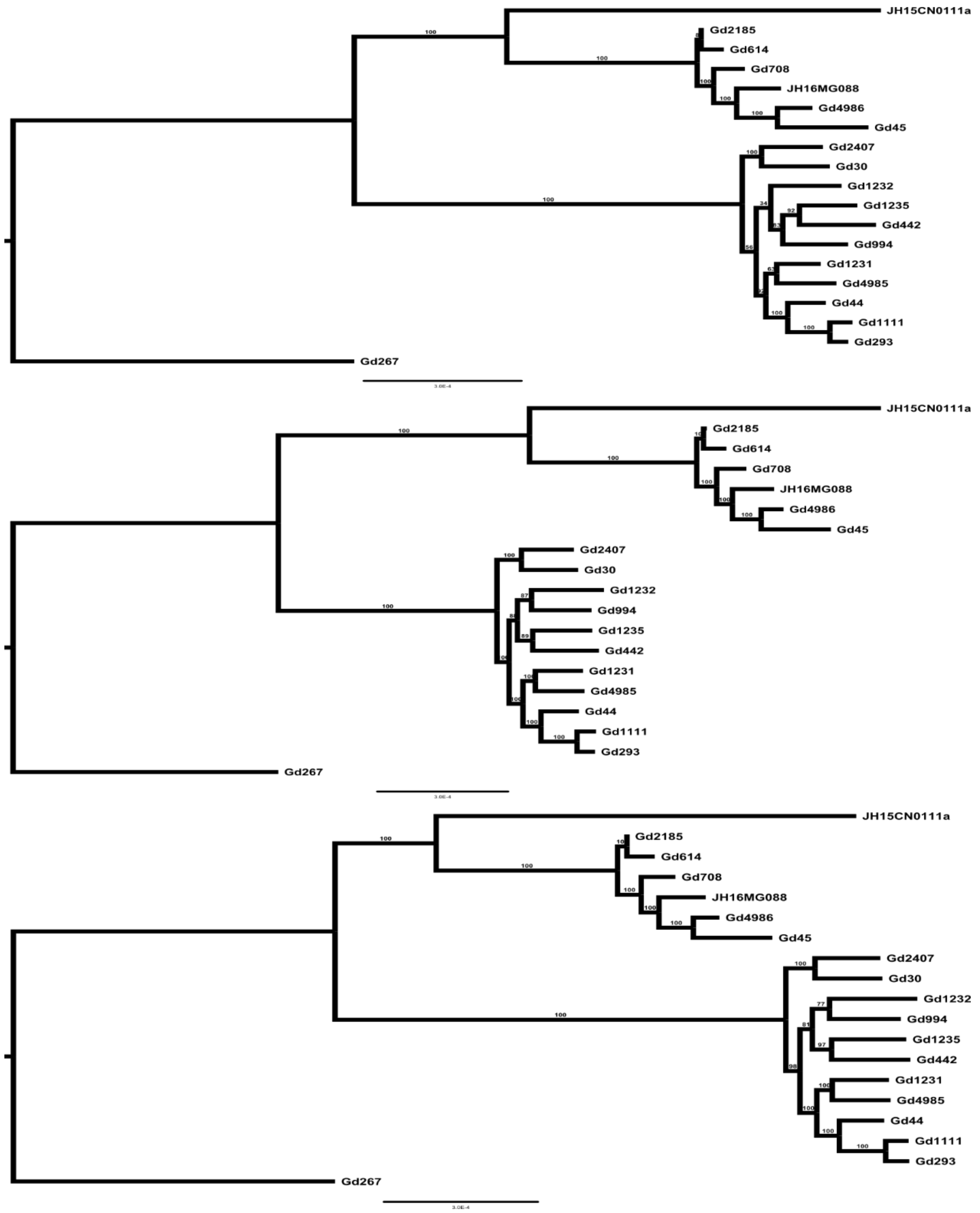

Fig. S5. Phylogenetic tree of different partitions with 1000 bootstraps as percentages. Tree for 662 BUSCO genes (total of 1,609,347 bp) when using Gd45 as the reference genome for mapping (top; see Figure 2b for results when mapping on Gd293), for 10 kb window partitions when using Gd293 (1,275 windows totalling 9,191,418 bp; gene concordance factor of 67.1 and 75.3 for *Pd*-1 &*Pd*-2 respectively; middle) or Gd45 (1,288 windows totalling 9,265,179bp; gene concordance factor of 81.4 and 41.3 for *Pd*-1 & *Pd*-2 respectively; bottom) as the reference genome for mapping. The branch to the outgroup has been shortened for visualisation purposes.

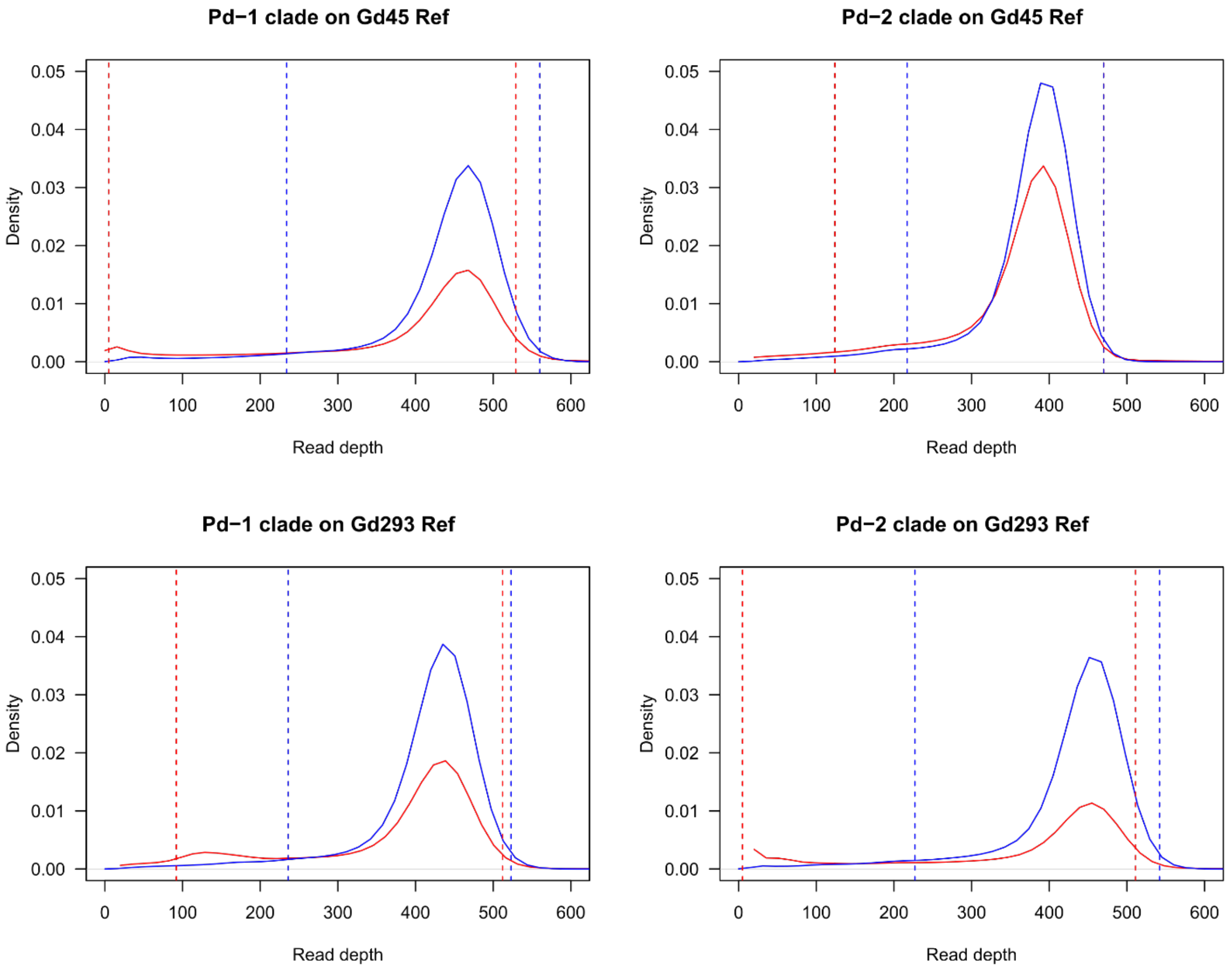

Fig. S6. Read depth for *Pd*-1 (left, 69 isolates) and *Pd*-2 (right, 63 isolates) clades when mapping the pool-seq data on Gd45 (top) and Gd293 (bottom) reference genomes. Density (continuous line) and 95% highest density interval (dashed vertical lines) are represented before (red) and after filtering (blue). See section 'Data filtering' in the Methods for details.

Supplementary Tables S4 to S14

Table S1– (Provided as separate Excel file, first sheet in "41586_2025_9060_MOESM5_ESM.xlsx")

Raw data and metadata. Provides the genotypic raw data (alleles) for the 18 microsatellite loci of all used isolates with corresponding information on sampling site, clade (*Pd*-1 / *Pd*-2), substrate (bat / environment / caving gear), bat species identity (if sample was collected from / close to a bat) and mating type of the fungal isolates. Furthermore, an overview is provided over which isolates were used for which analyses (regarding the genotypic data and expansion rate analysis).

Table S2– (Provided as separate Excel file, second sheet in "41586_2025_9060_MOESM5_ESM.xlsx")

Summary by country. Summary on the genotypic dataset by country. We provide information on the number of sites from which *P. destructans* isolates were obtained, the number of swab samples, the number of cultures and the number of unique multi-locus genotypes per country. Furthermore, we provide the percentages of isolates belonging to clade *Pd*-1 and *Pd*-2 (out of all isolates), the number of isolates obtained from bats and hibernacula environments as well as corresponding bat species composition for isolates from bats by country.

Table S3– (Provided as separate Excel file, third sheet in "41586_2025_9060_MOESM5_ESM.xlsx")

Summary by site. Summary on the genotypic dataset, abiotic (temperature and humidity), and biotic (bat species composition) factors by site. We provide information on the number of swab samples from which *P. destructans* isolates were obtained, the number of isolates and the number of unique multi-locus genotypes per site. Furthermore, by site, we provide the percentages of isolates belonging to clade *Pd*-1 and *Pd*-2 (out of all isolates), the number of isolates obtained from bats and hibernacula environments as well as corresponding bat species composition from which isolates were collected from. For *M. daubentonii*, we also provide data on the presence/absence of the species at that site.

Table S4– (Provided as separate Excel file, fourth sheet in "41586_2025_9060_MOESM5_ESM.xlsx")

DAPC results. Posterior probability values of the assignment of the 33 North-American isolates to each of the European sites (excluding the site in the Ural Mountains) to determine the most likely source population of the North American introduction. For further information on the analyses, see section 'Analyses of multi-locus genotypes' in the Methods.

Table S5– (Provided as separate Excel file, fifth sheet in "41586_2025_9060_MOESM5_ESM.xlsx")

SPASIBA results. Likelihood of each grid cell being the source population of the North American introduction (averaged over the 33 North American isolates). The grid used for Europe covers 198 cells in longitude (as rows in the table) and 120 cells in latitude (as columns in the table).

Table S6.

Allelic richness (number of different alleles) for each of the microsatellite markers used in the study overall and for each of the clades separately. For each locus, we used BLAST to determine its location on the Gd293 reference genome, demonstrating that the loci are distributed across 14 different contigs and thus cover the majority of the genome's 18 contigs (see Table S12). Only the two longest contigs (4.4 & 3.8 Mb) harbour more than one locus.

Locus	Allelic richness overall	Allelic richness *Pd*-1	Allelic richness *Pd*-2	Position on Gd293 reference genome
**Pd1**	61	49	23	contig_40
**Pd2**	86	86	8	contig_33
**Pd3**	36	35	5	contig_30
**Pd4**	50	42	10	contig_28
**Pd5**	93	71	40	contig_34
**Pd6**	10	10	2	contig_39
**Pd7**	55	55	7	contig_10
**Pd9**	11	11	7	contig_33
**Pd10**	15	15	1	contig_34
**Pd11**	38	38	5	contig_48
**Pd12**	28	11	20	contig_23
**Pd13**	25	25	8	contig_26
**Pd14**	27	27	1	contig_18
**Pd17**	14	14	2	contig_11
**Pd19**	30	21	22	contig_37
**Pd21**	13	13	5	contig_15
**Pd22**	49	44	5	contig_34
**Pd23**	30	30	6	contig_34
Mean	37.3	33.2	9.8	

Table S7.

Coefficients and convergence statistics from the Bayesian hierarchical model testing for an association between bat species and fungal clade identity (in Europe). The data were analysed with a binomial distribution and probit link including site and samples (nested within sites) as group level effects, and bat species, latitude and longitude as population-level effects (see section 'Statistical analyses' in the Methods). For each isolate, the response variable was the clade identity of the fungus (*Pd*-1: 0; *Pd*-2: 1; total of 4,295 isolates). The estimation for each bat species (Mdas: *M. dasycneme*, Mdau: *M. daubentonii*, Mmb: *M. myotis/blythii*, Mnec: *M. nattereri/escalerai/crypticus*) was parameterized in relation to the baseline category "Mmys" (=*M. mystacinus*) and the parameters estimated therefore represent the difference from *M. mystacinus*, incorporating the effect of samples, sites, latitude, and longitude. Est. Error: estimated standard error for the estimate; l-95% CI and u-95% CI, lower and upper (respectively) credible interval of the estimate; Rhat: potential scale reduction factor on split chains; Bulk_ESS and Tail_ESS: effective sample size for bulk and tail samples respectively.
The model converged as denoted by the Rhat values of 1 for all estimated parameters and the Bulk_ESS and Tail_ESS values >10,000. All GVIF values were below 1.77, suggesting very limited collinearity between explanatory variables. Scaled latitude (estimate [95% credible intervals]: 4.67 [-1.42– 13.39]) and longitude (1.96 [-2.18 – 7.07]) had no effect as illustrated by their credible interval overlapping with zero. Regarding bat species, the baseline group, *Myotis mystacinus* had a log odds ratio 95% credible interval spanning zero (-18.03 – 17.08), meaning that the species is equally likely to be infected by *Pd*-1 or *Pd*-2. Other bat species differed markedly from the baseline group; namely, *M. dasycneme*, *M. myotis/blythii* and *M. nattereri/escalerai/crypticus*, which all had negative log odd ratios (and their 95% credible intervals not overlapping with zero), were less likely to harbour *Pd*-2 than *Pd*-1. When converting the log odd ratios to probabilities, the 95% credible interval of the estimated probability of different bats species to harbour *Pd*-2 were 1.07×10^{-42} – 4.98×10^{-5} for *M. dasycneme*, 8.07×10^{-26} – 1.65×10^{-5} for *M. myotis/blythii*, and 2.38×10^{-22} – 0.017 for *M. nattereri/escalerai/crypticus*. The pattern was the opposite for *M. daubentonii* with a positive log odd ratio (and a positive 95% credible interval), meaning that the species is more likely to harbour *Pd*-2 compared to *M. mystacinus*. Indeed, the 95% credible interval of the estimated probability of *M. daubentonii* to harbour *Pd*-2 was ≈ 1.00 for both, the lower and upper bound of the 95% credible interval. All together, these results demonstrate that the probability of encountering *Pd*-1 or *Pd*-2 is not related to the latitude/longitude but is directly affected by the host bat species.

	Estimate	Est. Error	l-95% CI	u-95% CI	Rhat	Bulk_ESS	Tail_ESS
Intercept (Mmys)	-0.74	8.77	-18.03	17.08	1	25336	25383
Mdas	-41.43	22.38	-96.64	-9.91	1	16078	17231
Mdau	1097.52	374.02	530.45	1965.03	1	12063	11403
Mmb	-28.61	12.05	-57.78	-11.01	1	13475	14484
Mnec	-21.6	11.67	-49.79	-4.02	1	14207	14546
Lat_scaled	4.67	3.73	-1.42	13.39	1	17055	19565
Lon_scaled	1.96	2.31	-2.18	7.07	1	19275	21898

Table S8.

Pixel density from photos taken of *P. destructans* cultures one week (seven days) and eight weeks (56 days) after culturing and storage at 15°C. For each photo the pixel density was extracted for three equally sized rectangles, each neither touching the visible culture nor the edge of the petri dish and the median value was used to visualise the change in pixel density (Extended Data Figure 2; see also section 'Analysis of the culture darkness' in the Methods for further information).

Culture name	Clade	After 7 days				After 56 days			
		Rect 1	Rect 2	Rect 3	Median	Rect 1	Rect 2	Rect 3	Median
Gd_00030-2aa	*Pd*-1	0.8980	0.9176	0.9098	0.9098	0.9098	0.9333	0.9020	0.9098
Gd_00048-ca	*Pd*-1	0.8824	0.9137	0.8902	0.8902	0.9020	0.9255	0.8902	0.9020
Gd_00161-dgb	*Pd*-1	0.8902	0.9176	0.8980	0.8980	0.9020	0.9333	0.9020	0.9020
Gd_00185-abea	*Pd*-1	0.8745	0.9020	0.8863	0.8863	0.8902	0.9137	0.8941	0.8941
Gd_00194-a2ab	*Pd*-1	0.8784	0.9137	0.8941	0.8941	0.9059	0.9333	0.9059	0.9059
Gd_00198-bd	*Pd*-1	0.8667	0.8980	0.8745	0.8745	0.8941	0.9137	0.8824	0.8941
Gd_00281-aad	*Pd*-1	0.8902	0.9176	0.8902	0.8902	0.9176	0.9373	0.9059	0.9176
Gd_00559-ab	*Pd*-1	0.8941	0.9176	0.8941	0.8941	0.9216	0.9529	0.9137	0.9216
Gd_00759-aa	*Pd*-1	0.8824	0.9059	0.8824	0.8824	0.9020	0.9176	0.8863	0.9020
Gd_00819-ceaa	*Pd*-1	0.8784	0.9059	0.8824	0.8824	0.9137	0.9333	0.9098	0.9137
Gd_00886-aa	*Pd*-1	0.8824	0.9059	0.8863	0.8863	0.9137	0.9294	0.9098	0.9137
Gd_01038-bd	*Pd*-1	0.8902	0.9137	0.8863	0.8902	0.9412	0.9569	0.9647	0.9569
Gd_01064-aa	*Pd*-1	0.8745	0.9020	0.8824	0.8824	0.9216	0.9529	0.9569	0.9529
Gd_01077-bb	*Pd*-1	0.8824	0.9059	0.8824	0.8824	0.9216	0.9373	0.9451	0.9373
Gd_01094-bb	*Pd*-1	0.8902	0.9137	0.8980	0.8980	0.8980	0.9294	0.8980	0.8980
Gd_01095-ac	*Pd*-1	0.8902	0.9137	0.9020	0.9020	0.9059	0.9333	0.9059	0.9059
Gd_01144-ba	*Pd*-1	0.8824	0.9098	0.8863	0.8863	0.9059	0.9255	0.8941	0.9059
Gd_01244-aca	*Pd*-1	0.8863	0.9098	0.8941	0.8941	0.9020	0.9373	0.9059	0.9059
Gd_01248-aca	*Pd*-1	0.8941	0.9176	0.8980	0.8980	0.9020	0.9294	0.8941	0.9020
Gd_01253-acb	*Pd*-1	0.8745	0.9059	0.8745	0.8745	0.8941	0.9137	0.8863	0.8941
Gd_01719-ac	*Pd*-1	0.8824	0.9137	0.8902	0.8902	0.9098	0.9373	0.9020	0.9098
Gd_01770-aa	*Pd*-1	0.8902	0.9176	0.8941	0.8941	0.9176	0.9412	0.9137	0.9176
Gd_01794-ab	*Pd*-1	0.8706	0.9020	0.8863	0.8863	0.8902	0.9216	0.8902	0.8902
Gd_01880-aa	*Pd*-1	0.8706	0.8941	0.8745	0.8745	0.9059	0.9255	0.8980	0.9059
Gd_01882-ad	*Pd*-1	0.8784	0.9137	0.9020	0.9020	0.9020	0.9255	0.8980	0.9020
Gd_01952-af	*Pd*-1	0.8706	0.8980	0.8745	0.8745	0.9020	0.9216	0.8902	0.9020
Gd_01990-ad	*Pd*-1	0.8980	0.9176	0.9020	0.9020	0.9098	0.9373	0.9059	0.9098
Gd_01992-ad	*Pd*-1	0.8824	0.9059	0.8980	0.8980	0.8980	0.9333	0.9059	0.9059
Gd_02008-aaaa	*Pd*-1	0.8627	0.8941	0.8784	0.8784	0.9059	0.9333	0.9059	0.9059
Gd_02032-aa	*Pd*-1	0.8627	0.8941	0.8706	0.8706	0.8784	0.9137	0.8824	0.8824
Gd_02330-ab	*Pd*-1	0.8627	0.9020	0.8745	0.8745	0.9020	0.9373	0.9020	0.9020
Gd_02392-aa	*Pd*-1	0.8745	0.9059	0.8863	0.8863	0.8902	0.9176	0.8902	0.8902
Gd_02455-aaa	*Pd*-1	0.8667	0.9020	0.8627	0.8667	0.8863	0.9137	0.8863	0.8863
Gd_02465-ac	*Pd*-1	0.8627	0.8980	0.8627	0.8627	0.8941	0.9216	0.8784	0.8941
Gd_02472-ab	*Pd*-1	0.8745	0.9255	0.8941	0.8941	0.8863	0.9216	0.8980	0.8980
Gd_02473-aa	*Pd*-1	0.8745	0.9059	0.8784	0.8784	0.8980	0.9255	0.8902	0.8980
Gd_02486-aa	*Pd*-1	0.8784	0.9098	0.8824	0.8824	0.8980	0.9294	0.8980	0.8980
Gd_02496-aa	*Pd*-1	0.8667	0.8980	0.8627	0.8667	0.9059	0.9333	0.9020	0.9059
Gd_02501-aa	*Pd*-1	0.8627	0.8980	0.8745	0.8745	0.8980	0.9333	0.9020	0.9020
Gd_02601-aa	*Pd*-1	0.8784	0.8980	0.8745	0.8784	0.8863	0.9020	0.8863	0.8863

Culture name	Clade	After 7 days				After 56 days			
		Rect 1	Rect 2	Rect 3	Median	Rect 1	Rect 2	Rect 3	Median
Gd_02702-ae	*Pd*-1	0.8745	0.8980	0.8745	0.8745	0.8941	0.9216	0.8863	0.8941
Gd_04129-ca	*Pd*-1	0.8706	0.9020	0.8863	0.8863	0.8902	0.9255	0.8941	0.8941
Gd_04994-cb	*Pd*-1	0.8824	0.9059	0.8941	0.8941	0.9020	0.9176	0.8980	0.9020
Gd_05149-cb	*Pd*-1	0.8824	0.9255	0.8980	0.8980	0.8980	0.9255	0.8980	0.8980
Gd_05163-ca	*Pd*-1	0.8941	0.9137	0.8824	0.8941	0.8980	0.9176	0.8824	0.8980
Gd_00518-cb	*Pd*-2	0.9216	0.9412	0.9059	0.9216	0.8941	0.9216	0.8863	0.8941
Gd_00614-baa	*Pd*-2	0.9373	0.9529	0.9255	0.9373	0.8941	0.9098	0.8745	0.8941
Gd_00708-ba	*Pd*-2	0.9020	0.9294	0.8902	0.9020	0.8431	0.8706	0.8431	0.8431
Gd_00708-ca	*Pd*-2	0.9137	0.9412	0.9137	0.9137	0.8118	0.8706	0.8275	0.8275
Gd_00763-aa	*Pd*-2	0.9176	0.9490	0.9176	0.9176	0.8941	0.9176	0.8902	0.8941
Gd_00953-aa	*Pd*-2	0.9098	0.9412	0.9176	0.9176	0.8941	0.9255	0.8863	0.8941
Gd_00956-bc	*Pd*-2	0.9137	0.9373	0.9020	0.9137	0.8863	0.9137	0.8941	0.8941
Gd_01031-aa	*Pd*-2	0.9020	0.9294	0.9137	0.9137	0.8667	0.9059	0.8627	0.8667
Gd_01056-adaaa	*Pd*-2	0.8941	0.9216	0.8980	0.8980	0.8627	0.9020	0.8549	0.8627
Gd_01089-ab	*Pd*-2	0.9137	0.9333	0.9059	0.9137	0.8118	0.8549	0.8235	0.8235
Gd_01249-aab	*Pd*-2	0.9020	0.9294	0.9020	0.9020	0.8510	0.8863	0.8549	0.8549
Gd_01370-ac	*Pd*-2	0.9059	0.9255	0.9059	0.9059	0.9020	0.9216	0.8941	0.9020
Gd_01416-aa	*Pd*-2	0.8902	0.9137	0.9020	0.9020	0.8706	0.8980	0.8745	0.8745
Gd_01424-aa	*Pd*-2	0.9059	0.9373	0.9216	0.9216	0.8941	0.9176	0.8941	0.8941
Gd_01514-ad	*Pd*-2	0.9176	0.9490	0.9137	0.9176	0.9020	0.9294	0.8941	0.9020
Gd_01561-aa	*Pd*-2	0.9098	0.9255	0.9020	0.9098	0.8941	0.9098	0.8784	0.8941
Gd_01600-aa	*Pd*-2	0.9294	0.9529	0.9255	0.9294	0.8824	0.9255	0.8275	0.8824
Gd_01600-ab	*Pd*-2	0.9137	0.9255	0.9020	0.9137	0.8941	0.9137	0.8902	0.8941
Gd_01604-ac	*Pd*-2	0.9098	0.9412	0.9176	0.9176	0.8902	0.9176	0.8902	0.8902
Gd_01636-ad	*Pd*-2	0.8824	0.9176	0.8863	0.8863	0.8667	0.8902	0.8706	0.8706
Gd_01657-ad	*Pd*-2	0.9020	0.9255	0.9059	0.9059	0.8706	0.8824	0.8784	0.8784
Gd_01813-ab	*Pd*-2	0.8902	0.9020	0.8863	0.8902	0.8824	0.8980	0.8627	0.8824
Gd_01815-ab	*Pd*-2	0.8980	0.9216	0.8980	0.8980	0.8510	0.8784	0.8549	0.8549
Gd_01844-db	*Pd*-2	0.9020	0.9294	0.9020	0.9020	0.8824	0.8941	0.8745	0.8824
Gd_01855-aa	*Pd*-2	0.9098	0.9373	0.9137	0.9137	0.8863	0.9137	0.8980	0.8980
Gd_01937-abb	*Pd*-2	0.8941	0.9294	0.9059	0.9059	0.9098	0.9255	0.9059	0.9098
Gd_01938-aca	*Pd*-2	0.9020	0.9294	0.9059	0.9059	0.8784	0.8941	0.8784	0.8784
Gd_02351-aa	*Pd*-2	0.9020	0.9294	0.8980	0.9020	0.8745	0.9020	0.8627	0.8745
Gd_02450-abaa	*Pd*-2	0.9059	0.9255	0.8980	0.9059	0.8196	0.8667	0.8353	0.8353
Gd_02475-ab	*Pd*-2	0.9098	0.9333	0.9020	0.9098	0.8863	0.9098	0.8863	0.8863
Gd_02506-ab	*Pd*-2	0.9020	0.9333	0.9059	0.9059	0.8902	0.9098	0.8941	0.8941
Gd_04986-cc	*Pd*-2	0.8824	0.9176	0.9020	0.9020	0.8627	0.8980	0.8745	0.8745
Gd_05012-ea	*Pd*-2	0.9176	0.9412	0.9176	0.9176	0.8706	0.9059	0.8706	0.8706
Gd_05021-cb	*Pd*-2	0.8941	0.9176	0.8902	0.8941	0.8392	0.8745	0.8549	0.8549

Table S9.

Colony expansion rates obtained from photos taken of *P. destructans* cultures one week, 3 weeks, 5 weeks and 7 weeks (49 days) after culturing with storage at 15°C. See section 'Analysis of growth' in the Methods for further information.

Culture name	Clade	Size week 1 [cm^3]	Size week 3 [cm^3]	Size week 5 [cm^3]	Size week 7 [cm^3]
Gd_00030-2aa	*Pd*-1	0.879	2.591	3.992	3.765
Gd_00048-ca	*Pd*-1	0.645	2.064	4.376	4.656
Gd_00161-dgb	*Pd*-1	0.627	2.061	3.833	4.454
Gd_00185-abea	*Pd*-1	0.538	1.898	3.648	3.833
Gd_00194-a2ab	*Pd*-1	0.338	1.136	2.496	2.903
Gd_00198-bd	*Pd*-1	0.646	0.708	1.214	4.245
Gd_00281-aad	*Pd*-1	0.891	2.135	4.043	4.227
Gd_00559-ab	*Pd*-1	0.283	0.957	2.301	2.941
Gd_00759-aa	*Pd*-1	0.223	0.896	2.276	3.107
Gd_00819-ceaa	*Pd*-1	0.457	1.549	3.112	3.918
Gd_00886-aa	*Pd*-1	0.857	2.889	4.871	4.831
Gd_01038-bd	*Pd*-1	0.407	1.287	2.730	3.461
Gd_01064-aa	*Pd*-1	0.749	2.433	4.266	4.261
Gd_01077-bb	*Pd*-1	0.532	1.967	4.268	4.502
Gd_01094-bb	*Pd*-1	0.773	2.400	4.305	4.553
Gd_01095-ac	*Pd*-1	0.701	2.468	4.467	4.222
Gd_01144-ba	*Pd*-1	0.674	2.183	4.254	4.668
Gd_01244-aca	*Pd*-1	0.768	2.512	4.461	4.637
Gd_01248-aca	*Pd*-1	0.913	2.915	4.436	4.401
Gd_01253-acb	*Pd*-1	0.511	1.714	3.687	4.612
Gd_01719-ac	*Pd*-1	0.385	1.428	2.851	3.610
Gd_01770-aa	*Pd*-1	0.387	1.567	3.194	4.018
Gd_01794-ab	*Pd*-1	0.508	0.627	0.838	0.850
Gd_01880-aa	*Pd*-1	0.373	1.070	2.175	3.132
Gd_01882-ad	*Pd*-1	0.462	1.702	3.428	3.756
Gd_01952-af	*Pd*-1	0.447	0.607	0.654	0.693
Gd_01990-ad	*Pd*-1	0.242	0.788	1.371	2.419
Gd_01992-ad	*Pd*-1	0.402	1.541	3.232	3.952
Gd_02008-aaaa	*Pd*-1	0.667	2.426	4.645	4.610
Gd_02032-aa	*Pd*-1	0.500	1.749	3.372	4.327
Gd_02330-ab	*Pd*-1	0.540	1.816	3.466	3.946
Gd_02392-aa	*Pd*-1	0.953	2.803	4.915	4.467
Gd_02455-aaa	*Pd*-1	0.245	0.738	1.446	1.425
Gd_02465-ac	*Pd*-1	1.424	3.848	5.468	5.584
Gd_02472-ab	*Pd*-1	0.690	2.221	4.190	4.548
Gd_02473-aa	*Pd*-1	0.260	0.416	0.615	0.703
Gd_02486-aa	*Pd*-1	0.616	2.246	3.981	4.354
Gd_02496-aa	*Pd*-1	0.735	1.659	2.833	3.558
Gd_02501-aa	*Pd*-1	0.195	0.391	0.628	0.977
Gd_02601-aa	*Pd*-1	0.795	2.496	4.739	5.500

Culture name	Clade	Size week 1 [cm^3]	Size week 3 [cm^3]	Size week 5 [cm^3]	Size week 7 [cm^3]
Gd_02702-ae	*Pd*-1	0.853	3.052	5.128	4.738
Gd_04129-ca	*Pd*-1	0.790	2.626	4.711	4.868
Gd_04994-cb	*Pd*-1	0.692	2.399	4.480	4.659
Gd_05149-cb	*Pd*-1	0.388	0.424	0.444	0.457
Gd_05163-ca	*Pd*-1	0.489	2.027	4.334	4.992
Gd_00518-cb	*Pd*-2	0.6521	2.2251	3.8270	3.8831
Gd_00614-baa	*Pd*-2	0.5567	0.6858	0.7191	0.7370
Gd_00708-ba	*Pd*-2	0.5996	0.6690	0.7001	0.7484
Gd_00708-ca	*Pd*-2	0.7576	1.0497	3.4190	5.7270
Gd_00763-aa	*Pd*-2	0.4200	1.7885	3.7882	3.8555
Gd_00953-aa	*Pd*-2	0.9133	2.9315	3.8727	3.8396
Gd_00956-bc	*Pd*-2	0.5478	1.9538	3.7081	4.0701
Gd_01031-aa	*Pd*-2	1.2911	3.7409	5.5759	5.2589
Gd_01056-adaaa	*Pd*-2	0.9179	3.0457	5.3966	5.5538
Gd_01089-ab	*Pd*-2	0.9359	3.0514	5.7115	5.6921
Gd_01249-aab	*Pd*-2	1.1728	3.5041	6.2390	6.2828
Gd_01370-ac	*Pd*-2	0.5972	2.0477	3.2581	2.7183
Gd_01416-aa	*Pd*-2	0.7588	2.5767	4.2034	4.0085
Gd_01424-aa	*Pd*-2	0.7529	2.3127	3.1263	2.7987
Gd_01514-ad	*Pd*-2	0.8713	2.6149	3.5135	3.4780
Gd_01561-aa	*Pd*-2	0.6221	2.0213	3.4294	3.4885
Gd_01600-aa	*Pd*-2	0.3209	0.9884	1.7585	2.3185
Gd_01600-ab	*Pd*-2	0.3934	1.1842	1.5940	1.5908
Gd_01604-ac	*Pd*-2	1.1228	2.9400	3.5750	3.3217
Gd_01636-ad	*Pd*-2	0.4983	1.6786	2.6397	2.6172
Gd_01657-ad	*Pd*-2	0.9339	2.8469	4.5663	4.6621
Gd_01813-ab	*Pd*-2	0.7496	2.1961	4.4178	4.7803
Gd_01815-ab	*Pd*-2	0.7887	2.5532	5.0978	5.1668
Gd_01844-db	*Pd*-2	0.6962	2.3702	4.2879	4.4505
Gd_01855-aa	*Pd*-2	0.9087	2.4767	3.4104	2.9910
Gd_01937-abb	*Pd*-2	0.5592	1.7213	3.0145	2.9392
Gd_01938-aca	*Pd*-2	0.3608	1.6871	3.3918	3.7549
Gd_02351-aa	*Pd*-2	0.2903	1.0638	2.3224	3.5478
Gd_02450-abaa	*Pd*-2	1.0653	3.2877	5.4639	5.4908
Gd_02475-ab	*Pd*-2	0.6273	2.1151	3.7461	3.7235
Gd_02506-ab	*Pd*-2	0.2878	0.8003	1.4372	1.9627
Gd_04986-cc	*Pd*-2	0.7415	2.6430	5.1924	4.8339
Gd_05012-ea	*Pd*-2	0.6855	2.3757	4.8085	4.4993
Gd_05021-cb	*Pd*-2	0.6546	0.7518	0.7803	0.8022

Table S10.

Information on sequences and their origin including the isolates' full name, country of origin, substrate they were recovered from (wall and bat with information on bat species), and Sequence Read Archive Number (SRA). Contaminants were found via irregularities in GC content and identified through BLAST database, leading to the exclusion of seven contigs (see section 'Genome assembly' in the Methods). CCF numbers refer to the accession number of culture obtained from the Culture Collection of Fungi, Prague, Czech Republic.
* SRA (Illumina followed by Oxford Nanopore Technologies) available in the BioProject no. PRJNA862744.

**Short Name**	**Clade**	**Full name**	**Country**	**From**	**SRA**
Gd293	*Pd*-1	Gd_00293-aad	Ukraine	Bat (*Myotis myotis*)	SRR30476787<br>SRR30476775
Gd442	*Pd*-1	Gd_00442-ba	Bulgaria	Bat (*M. myotis/M. blythii*)	SRR30476795<br>SRR30476783
Gd994	*Pd*-1	Gd_00994-aaa	Austria	Bat (*M. myotis*)	SRR30476771<br>SRR30476780
Gd1111	*Pd*-1	Gd_01111-aaa=20631-21 (type isolate)	USA	Bat (*M.lucifugus*)	SRR1952982<br>SRR30476772
Gd2407	*Pd*-1	Gd_02407-aa	France	Bat (*M. myotis*)	SRR30476769<br>SRR30476778
Gd4985	*Pd*-1	Gd_04985-ea=CCF-4985	Russia	Bat (*M. dasycneme*)	SRR30476768<br>SRR30476777
Gd1231	*Pd*-1	GU999986=Tesa-OT-8-09	Germany	Bat (*M. myotis*)	SRR6011467
Gd1232	*Pd*-1	GU350433=Tesa-OT-10-09	Switzerland	Bat (*M. myotis*)	SRR6011468
Gd1235	*Pd*-1	GU350434=Tesa-OT-14-09	Hungary	Bat (*M. myotis*)	SRR6011465
Gd30	*Pd*-1	Gd30	France	Bat (*M. myotis*)	SRR6011497
Gd44	*Pd*-1	Gd44	Ukraine	Bat (*M. myotis*)	SRR6011496
Gd45	*Pd*-2	Gd_00045-aaab	Germany	Bat (*Myotis daubentonii*)	SRR30476786<br>SRR30476774
Gd614	*Pd*-2	Gd_00614-baa	Spain	Wall	SRR30476784<br>SRR30476782
Gd708	*Pd*-2	Gd_00708-ba	Poland	Wall	SRR30476773<br>SRR30476781
Gd2185	*Pd*-2	Gd_02185-ab	Bulgaria	Wall	SRR30476770<br>SRR30476779
Gd4986	*Pd*-2	Gd_04986-cc=CCF-4986	Russia	Bat (*M. dasycneme*)	SRR30476767<br>SRR30476776
JH15CN0111a	*Pd*-2	JH15CN0111a	China	Bat (*M. petax*)	SRR6011485
JH16MG088	*Pd*-2	JH16MG088	Mongolia	Bat (*Plecotus ognevi*)	SRR6011486
Gd267	Out-group	Gd_00267-ac	Germany	Bat (*M. daubentonii*)	SRR30476796<br>SRR30476785

Table S11.

Statistics of the long-read sequences (Oxford Nanopore Technology) and the associated assembled genomes. For long-read sequencing, the N50 of the raw reads is presented along with the total amount of data generated (in Gb). Genome assembly statistics, including the number of contigs and length of genome, are noted after polishing and removal of contigs below 10,000 base pairs in length. Complete BUSCO is presented (there are 758 Fungal genes in the v10 orthoDB database, see section 'Genome annotation' in the Methods). The isolate in bold denotes the outgroup used in the phylogeny. N50 and length data are presented in base pairs (bp).

	Sequences		Genomes assemblies						
**Isolate**	**N50**	**Gb**	**BUSCO gene number**	**N50**	**N. contigs**	**Length**	**Number of repeats in library**	**Length of repeats in library**	**% of repeats**
1111	3809	2.1	745	2,177,740	29	34,598,246	698	698,241	37.07
994	4144	2.2	746	1,810,452	38	34,860,382	701	698,898	36.89
442	2644	15.2	746	1,442,842	53	37,097,739	784	695,809	35.27
4986	1682	5.6	747	616,158	132	40,418,191	870	786,120	36.14
4985	5638	4.9	745	1,943,489	61	38,708,551	782	721,395	36.64
614	3613	1.4	745	1,855,880	42	37,190,968	707	709,228	40.67
708	2844	3.8	745	630,186	99	35,092,322	717	658,606	36.15
2407	4806	4.1	746	2,184,225	35	35,426,190	683	697,985	37.35
2185	4075	6.3	745	2,649,086	27	37,296,702	722	691,397	39.1
293	9189	7.1	744	2,399,949	18	38,552,003	797	794,881	37.19
45	3303	5.2	747	2,271,831	29	36,800,457	710	679,033	38.96
**267**	2919	3.3	**749**	**2,204,431**	**18**	**31,995,477**	**109**	**123,601**	**3.7**

Table S12.

Statistics used to identify contigs that likely contain repetitive sequences and/or contain large amount of missing data. * denotes contigs removed. These data originated from the mapping of Illumina reads of 18 isolates onto each of the two reference genomes, Gd293 and Gd45.

Reference genome	Contig ID	Mean no. isolate with missing data per site	Contig length	Mean depth
Gd293	contig_16	0.5	69874	231
Gd293	contig_48	1.5	227501	242
Gd293	contig_15	0.4	1158735	236
Gd293	contig_28	0.7	1451360	236
Gd293	contig_18	0.8	1512577	239
Gd293	contig_30	1.6	1811777	235
Gd293	contig_31	0.7	1888460	240
Gd293	contig_11	0.6	1935455	237
Gd293	contig_37	0.7	2017563	236
Gd293	contig_26	0.8	2160009	245
Gd293	contig_23	0.7	2196378	239
Gd293	contig_10	0.6	2207567	236
Gd293	contig_40	0.5	2399949	239
Gd293	contig_39	1	2841646	244
Gd293	contig_19	0.5	2985168	237
Gd293	contig_44*	13	3547778	341
Gd293	contig_33	0.7	3787825	238
Gd293	contig_34	0.6	4352381	235
Gd45	contig_317*	18	12775	0
Gd45	contig_281*	18	13433	0
Gd45	contig_316*	12.5	20011	2841
Gd45	contig_285*	1.8	20816	254
Gd45	contig_269*	9.6	22124	69
Gd45	contig_205*	14.2	25271	91
Gd45	contig_294*	8.1	25422	251
Gd45	contig_227*	1.5	32190	216
Gd45	contig_309	0.7	89485	240
Gd45	contig_242	0.4	322332	242
Gd45	contig_144	7.8	597633	295
Gd45	contig_219	1.2	792010	239
Gd45	contig_57	0.5	920655	237
Gd45	contig_225	0.4	1080045	244
Gd45	contig_163	2.9	1092458	267
Gd45	contig_158	1.1	1117255	243
Gd45	contig_231	0.4	1318045	239
Gd45	contig_252	2.5	1325870	245
Gd45	contig_116	0.8	1746838	240
Gd45	contig_24	0.5	2139374	281
Gd45	contig_131	1.3	2140546	244
Gd45	contig_179	0.7	2184625	243
Gd45	contig_61	1	2271831	240
Gd45	contig_235	1.7	2277293	248
Gd45	contig_198	0.7	2295305	240
Gd45	contig_78	0.5	2452036	240
Gd45	contig_208	1	2659243	245
Gd45	contig_104	1	3064491	241
Gd45	contig_82	1.3	4741045	246

Table S13.

Statistics for Illumina reads mapping to the reference genome from each clade, *Pd*-1 (Gd293) and *Pd*-2 (Gd45) calculated using vcftools. These statistics were calculated after filtering as detailed in 3.3.1. Npos covered: number of positions with a genotype (see 3.4.3). % covered: percentage of non-filtered positions that have a genotype.

	Ref Gd293; 21,609,224 non-filtered positions (=kept positions)			Ref Gd45; 22,041,192 non-filtered positions (=kept positions)		
**Short Name**	**Npos covered**	**Avg read depth**	**% covered**	**Npos covered**	**Avg read depth**	**% covered**
Gd30	20912440	76	96.8	20233448	76	91.8
Gd44	20768813	70	96.1	20181966	71	91.6
Gd293	21561825	947	99.8	20754289	927	94.2
Gd442	21099015	218	97.6	20595268	221	93.4
Gd994	21105501	166	97.7	20440973	169	92.7
Gd1111	21143022	179	97.8	20384228	183	92.5
Gd1231	20051756	85	92.8	19404235	87	88
Gd1232	20819401	131	96.3	20125174	132	91.3
Gd1235	20748056	129	96	20107784	130	91.2
Gd2407	21256829	199	98.4	20644597	200	93.7
Gd4985	21176578	193	98	20676248	197	93.8
Gd45	20692664	1018	95.8	22017017	1052	99.9
Gd614	20375114	129	94.3	21020760	134	95.4
Gd708	20624609	216	95.4	21587625	220	97.9
Gd2185	20478768	234	94.8	21464779	234	97.4
Gd4986	20571178	148	95.2	21658810	155	98.3
JH16MG088	20125179	58	93.1	21293931	59	96.6
JH15CN0111a	19831327	72	91.8	20031584	76	90.9

Table S14.

Summary of the accession numbers for Genbank sequences that were used to diagnose isolates of *Pd*-2 clade based on TEF1a and RPB2 genes' sequence.

Accession No. TEF1a	Accession No. RPB2	Country of origin	Reference
LN871350		Czech Republic	Zukal et al. 2016[18]
LN871344		Czech Republic	Zukal et al. 2016[18]
LN871365		Czech Republic	Zukal et al. 2016[18]
LR736723		Portugal	Sharma et al. 2019[19]
LR736724		Portugal	Sharma et al. 2019[19]
LR736725		Portugal	Sharma et al. 2019[19]
LR736726		Portugal	Sharma et al. 2019[19]
LR736727		Portugal	Sharma et al. 2019[19]
LR736728		Portugal	Sharma et al. 2019[19]
LR736729		Portugal	Sharma et al. 2019[19]
LR736732		Portugal	Sharma et al. 2019[19]
LR736733		Portugal	Sharma et al. 2019[19]
LR736734		Portugal	Sharma et al. 2019[19]
LR736735		Portugal	Sharma et al. 2019[19]
LR736736		Portugal	Sharma et al. 2019[19]
LR736737		Portugal	Sharma et al. 2019[19]
LR736738		Portugal	Sharma et al. 2019[19]
LR736739		Portugal	Sharma et al. 2019[19]
OP450961	OP450959	South Korea	Kim et al. 2022[20]
OP450962	OP450960	South Korea	Kim et al. 2022[20]

Table S15.

Information on samples used in the PoolSeq, including the isolates' full name, country of origin, substrate they were recovered from (wall and bat with information on bat species) as well as info on pooling before sequencing (4 sub-pools per clade). CCF numbers refer to the accession number of isolates obtained from the Culture Collection of Fungi, Prague, Czech Republic. SRA for Pd1_1 until Pd1_4 (SRR30476791, SRR30476790, SRR30476789, SRR30476788) and Pd2_1 until Pd2_4 (SRR30476766, SRR30476794, SRR30476793, SRR30476792) are available in the BioProject no. PRJNA862744.

No.	Clade	Full name	Country	From	Pool
1	*Pd*-1	Gd_00015-l2aea	Luxembourg	Bat (*Myotis myotis*)	Pd1_3
2	*Pd*-1	Gd_00026-2aeaaa	France	Bat (*M. myotis*)	Pd1_3
3	*Pd*-1	Gd_00030-2aaaa	France	Bat (*M. myotis*)	Pd1_1
4	*Pd*-1	Gd_00048-caaa	France	Bat (*Myotis mystacinus*)	Pd1_2
5	*Pd*-1	Gd_00085-baa	Germany	Bat (*M. myotis*)	Pd1_2
6	*Pd*-1	Gd_00161-dgbaa	Bulgaria	Wall	Pd1_3
7	*Pd*-1	Gd_00177-accaaa	Austria	Bat (*M. myotis*)	Pd1_3
8	*Pd*-1	Gd_00185-abeaa	Germany	Bat (*M. myotis*)	Pd1_3
9	*Pd*-1	Gd_00194-a2abaa	Netherlands	Bat (*Myotis dasycneme*)	Pd1_1
10	*Pd*-1	Gd_00198-bdac	Germany	Bat (*M. myotis*)	Pd1_4
11	*Pd*-1	Gd_00221-bbaa	Croatia	Wall	Pd1_1
12	*Pd*-1	Gd_00281-aadaa	Ukraine	Bat (*M. myotis*)	Pd1_2
13	*Pd*-1	Gd_00537-adaa	Germany	Bat (*M. myotis*)	Pd1_1
14	*Pd*-1	Gd_00559-abaa	Germany	Bat (*M. myotis*)	Pd1_3
15	*Pd*-1	Gd_00562-bbaa	Germany	Bat (*M. myotis*)	Pd1_2
16	*Pd*-1	Gd_00593-ccaa	Germany	Bat (*M. myotis*)	Pd1_2
17	*Pd*-1	Gd_00619-baaa	Poland	Wall	Pd1_3
18	*Pd*-1	Gd_00630-blaa	Sweden	Bat (*Myotis brandtii*)	Pd1_4
19	*Pd*-1	Gd_00642-aeaa	Hungary	Wall	Pd1_1
20	*Pd*-1	Gd_00661-bdaa	Germany	Wall	Pd1_1
21	*Pd*-1	Gd_00709-aaaaa	Poland	Wall	Pd1_2
22	*Pd*-1	Gd_00759-aaac	Poland	Bat (*M. myotis*)	Pd1_4
23	*Pd*-1	Gd_00819-ceaa	Germany	Wall	Pd1_3
24	*Pd*-1	Gd_00886-aaaa	Italy	Wall	Pd1_2
25	*Pd*-1	Gd_01038-bdaa	Slovakia	Bat (*M. myotis*)	Pd1_4
26	*Pd*-1	Gd_01064-aaaa	Hungary	Bat (*M. myotis*)	Pd1_1
27	*Pd*-1	Gd_01077-bbaa	France	Bat (*M. myotis*)	Pd1_2
28	*Pd*-1	Gd_01094-bbaa	France	Bat (*M. myotis*)	Pd1_1
29	*Pd*-1	Gd_01095-acaa	France	Bat (*M. myotis*)	Pd1_1
30	*Pd*-1	Gd_01144-baaa	Switzerland	Bat (*M. myotis*)	Pd1_2
31	*Pd*-1	Gd_01148-bcaaa	Hungary	Bat (*Myotis blythii*)	Pd1_2
32	*Pd*-1	Gd_01244-acaab	Belgium	Bat (*M. mystacinus*)	Pd1_2
33	*Pd*-1	Gd_01248-acaaa	Germany	Bat (*M. myotis*)	Pd1_4
34	*Pd*-1	Gd_01253-acbaa	Ukraine	Bat (*M. myotis*)	Pd1_2
35	*Pd*-1	Gd_01393-abaa	Germany	Bat (*M. myotis*)	Pd1_4

No.	Clade	Full name	Country	From	Pool
36	*Pd*-1	Gd_01719-acaa	Poland	Bat (*M. myotis*)	Pd1_3
37	*Pd*-1	Gd_01770-aaaa	Germany	Bat (*M. myotis*)	Pd1_3
38	*Pd*-1	Gd_01794-abaa	Germany	Bat (*M. myotis*)	Pd1_4
39	*Pd*-1	Gd_01880-aaaa	Germany	Bat (*M. myotis*)	Pd1_4
40	*Pd*-1	Gd_01882-adaa	Germany	Bat (*M. myotis*)	Pd1_1
41	*Pd*-1	Gd_01933-aaacaaa	Finland	Bat (*M. brandtii*)	Pd1_1
42	*Pd*-1	Gd_01952-afaa	France	Bat (*M. myotis*)	Pd1_4
43	*Pd*-1	Gd_01990-adaa	Romania	Bat (*M. myotis/M. blythii*)	Pd1_2
44	*Pd*-1	Gd_01992-adaa	Romania	Bat (*M. myotis/M. blythii*)	Pd1_3
45	*Pd*-1	Gd_02008-aaaaac	Republic of Moldova	Bat (*M. blythii*)	Pd1_4
46	*Pd*-1	Gd_02032-aaaa	Romania	Bat (*M. myotis/M. blythii*)	Pd1_4
47	*Pd*-1	Gd_02056-aaaaa	Slovakia	Bat (*M. myotis*)	Pd1_1
48	*Pd*-1	Gd_02330-abaa	France	Bat (*M. myotis*)	Pd1_2
49	*Pd*-1	Gd_02392-aaaa	France	Bat (*M. myotis*)	Pd1_3
50	*Pd*-1	Gd_02441-aaaaa	France	Bat (*M. myotis*)	Pd1_2
51	*Pd*-1	Gd_02455-aaaac	France	Bat (*M. myotis/M. blythii*)	Pd1_4
52	*Pd*-1	Gd_02465-acaa	France	Bat (*M. myotis*)	Pd1_4
53	*Pd*-1	Gd_02472-abaa	Poland	Bat (*M. myotis*)	Pd1_2
54	*Pd*-1	Gd_02473-aaaa	France	Bat (*M. myotis*)	Pd1_4
55	*Pd*-1	Gd_02486-aaaa	France	Bat (*M. myotis*)	Pd1_3
56	*Pd*-1	Gd_02496-aaaa	Denmark	Bat (*M. dasycneme*)	Pd1_3
57	*Pd*-1	Gd_02501-aaab	Denmark	Bat (*M. dasycneme*)	Pd1_1
58	*Pd*-1	Gd_02601-aaaa	Austria	Bat (*M. myotis*)	Pd1_2
59	*Pd*-1	Gd_02627-aca	Estonia	Bat (*M. brandtii*)	Pd1_1
60	*Pd*-1	Gd_02638-aaa	Sweden	Bat (*Myotis nattereri*)	Pd1_2
61	*Pd*-1	Gd_02648-abb	Latvia	Bat (*Plecotus auritus*)	Pd1_1
62	*Pd*-1	Gd_02702-aeaa	Bulgaria	Bat (*M. blythii*)	Pd1_1
63	*Pd*-1	Gd_02746-afaa	Bulgaria	Bat (*M. blythii*)	Pd1_4
64	*Pd*-1	Gd_02915-aaa	Germany	Bat (*M. myotis*)	Pd1_1
65	*Pd*-1	Gd_04129-caaa=CCF-4129	Czech Republic	Bat (*M. myotis*)	Pd1_4
66	*Pd*-1	Gd_04994-cbab=CCF-4994	Czech Republic	Bat (*M. myotis*)	Pd1_3
67	*Pd*-1	Gd_05003-daaa=CCF-5003	Czech Republic	Bat (*M. myotis*)	Pd1_3
68	Pd-1	Gd_05149-cbaa=CCF-5149	Czech Republic	Wall	Pd1_4
69	Pd-1	Gd_05163-caaa=CCF-5163	Czech Republic	Bat (*M. myotis*)	Pd1_3

No.	Clade	Full name	Country	From	Pool
1	*Pd*-2	Gd_00045-a2abea	Germany	Bat (*Myotis daubentonii*)	Pd2_1
2	*Pd*-2	Gd_00164-bacaaaa	England	Bat (*M. daubentonii*)	Pd2_4
3	*Pd*-2	Gd_00259-acbaa	Germany	Bat (*M. mystacinus*)	Pd2_1
4	*Pd*-2	Gd_00436-baaa	Bulgaria	Bat (*M. myotis/M. blythii*)	Pd2_4
5	*Pd*-2	Gd_00518-cbaaa	Germany	Bat (*M. nattereri*)	Pd2_1
6	*Pd*-2	Gd_00528-beaaa	Hungary	Bat (*M. myotis*)	Pd2_2
7	*Pd*-2	Gd_00539-abaa	Germany	Bat (*M. myotis*)	Pd2_3
8	*Pd*-2	Gd_00539-acaa	Germany	Bat (*M. myotis*)	Pd2_1
9	*Pd*-2	Gd_00573-aaaa	Poland	Bat (*M. myotis*)	Pd2_3
10	*Pd*-2	Gd_00614-baacaaa	Spain	Wall	Pd2_3
11	*Pd*-2	Gd_00708-baaaa	Poland	Wall	Pd2_3
12	*Pd*-2	Gd_00708-caaab	Poland	Wall	Pd2_2
13	*Pd*-2	Gd_00763-aaabb	Poland	Bat (*M. nattereri*)	Pd2_1
14	*Pd*-2	Gd_00785-abaaa	Germany	Bat (*M. daubentonii*)	Pd2_3
15	*Pd*-2	Gd_00953-aaaa	Germany	Bat (*M. myotis*)	Pd2_1
16	*Pd*-2	Gd_00956-bcaa	Germany	Bat (*M. myotis*)	Pd2_2
17	*Pd*-2	Gd_01031-aaaa	Germany	Bat (*M. myotis*)	Pd2_3
18	*Pd*-2	Gd_01056-adaaaaa	Germany	Bat (*M. myotis*)	Pd2_2
19	*Pd*-2	Gd_01089-abab	France	Bat (*M. mystacinus*)	Pd2_2
20	*Pd*-2	Gd_01238-acaaaaa	Germany	Bat (*M. myotis*)	Pd2_2
21	*Pd*-2	Gd_01249-aabaa	Germany	Bat (*M. myotis*)	Pd2_3
22	*Pd*-2	Gd_01250-aaaaa	Germany	Bat (*M. nattereri*)	Pd2_2
23	*Pd*-2	Gd_01370-acaa	Germany	Bat (*M. nattereri*)	Pd2_3
24	*Pd*-2	Gd_01395-aaaa	Germany	Bat (*M. daubentonii*)	Pd2_2
25	*Pd*-2	Gd_01416-aaaaa	Germany	Bat (*M. daubentonii*)	Pd2_3
26	*Pd*-2	Gd_01424-aaaa	Germany	Bat (*M. nattereri*)	Pd2_1
27	*Pd*-2	Gd_01514-adab	Germany	Bat (*M. myotis*)	Pd2_1
28	*Pd*-2	Gd_01561-aaaa	Germany	Bat (*M. daubentonii*)	Pd2_2
29	*Pd*-2	Gd_01600-aaab	Denmark	Bat (*M. daubentonii*)	Pd2_2
30	*Pd*-2	Gd_01600-abaa	Denmark	Bat (*M. daubentonii*)	Pd2_1
31	*Pd*-2	Gd_01604-aaaaa	France	Bat (*M. myotis*)	Pd2_1
32	*Pd*-2	Gd_01604-acaaa	France	Bat (*M. myotis*)	Pd2_4
33	*Pd*-2	Gd_01636-adaa	Germany	Bat (*M. daubentonii*)	Pd2_4
34	*Pd*-2	Gd_01657-adaa	Belgium	Bat (*M. myotis*)	Pd2_1
35	*Pd*-2	Gd_01813-abaa	France	Bat (*M. myotis*)	Pd2_1
36	*Pd*-2	Gd_01815-abaa	France	Bat (*M. myotis*)	Pd2_2
37	*Pd*-2	Gd_01844-daaa	Spain	Wall	Pd2_3
38	*Pd*-2	Gd_01844-dbaa	Spain	Wall	Pd2_1
39	*Pd*-2	Gd_01854-acaa	Latvia	Bat (*M. daubentonii*)	Pd2_4
40	*Pd*-2	Gd_01855-aaaa	Latvia	Bat (*M. daubentonii*)	Pd2_3
41	*Pd*-2	Gd_01937-abbaa	Finland	Bat (*M. nattereri*)	Pd2_4

No.	Clade	Full name	Country	From	Pool
42	*Pd*-2	Gd_01938-acaaa	Finland	Bat (*M. daubentonii*)	Pd2_3
43	*Pd*-2	Gd_02185-acaa	Bulgaria	Bat *(M. blythii*)	Pd2_1
44	*Pd*-2	Gd_02351-aaaa	France	Bat *(Myotis alcathoe)*	Pd2_1
45	*Pd*-2	Gd_02450-abaaab	France	Bat (*Myotis capaccinii*)	Pd2_2
46	*Pd*-2	Gd_02475-abab	France	Bat (*M. myotis*)	Pd2_2
47	*Pd*-2	Gd_02475-acaaaa	France	Bat (*M. myotis*)	Pd2_3
48	*Pd*-2	Gd_02506-abaa	Denmark	Bat (*M. daubentonii*)	Pd2_3
49	*Pd*-2	Gd_02630-acaab	Sweden	Bat (unknown)	Pd2_4
50	*Pd*-2	Gd_02631-abaaa	Sweden	Bat (*M. nattereri*)	Pd2_4
51	*Pd*-2	Gd_02632-aaaab	Sweden	Bat (*M. mystacinus/M. brandtii*)	Pd2_4
52	*Pd*-2	Gd_02633-acaaa	Sweden	Bat (*M. nattereri*)	Pd2_4
53	*Pd*-2	Gd_02642-aaa	Latvia	Bat (*M. daubentonii*)	Pd2_2
54	*Pd*-2	Gd_02642-aba	Latvia	Bat (*M. daubentonii*)	Pd2_4
55	*Pd*-2	Gd_02643-aaa	Latvia	Bat (*M. daubentonii*)	Pd2_4
56	*Pd*-2	Gd_02644-aaa	Latvia	Bat (*M. daubentonii*)	Pd2_4
57	*Pd*-2	Gd_02646-aca	Latvia	Bat (*M. daubentonii*)	Pd2_3
58	*Pd*-2	Gd_02647-aaa	Latvia	Bat (*M. daubentonii*)	Pd2_2
59	*Pd*-2	Gd_02650-aaa	Norway	Bat (*M. mystacinus/M. brandtii*)	Pd2_4
60	*Pd*-2	Gd_02652-aaa	Norway	Bat (*Eptesicus nilssonii*)	Pd2_2
61	*Pd*-2	Gd_04993-daaa=CCF-4993	Czech Republic	Bat (*M. myotis*)	Pd2_3
62	*Pd*-2	Gd_05012-eaaaa=CCF-5012	Czech Republic	Bat (*M. myotis*)	Pd2_1
63	*Pd*-2	Gd_05021-cbaaa=CCF-5021	Czech Republic	Bat (*M. myotis*)	Pd2_4

Table S16.

Results of the Approximate Bayesian Computation method testing alternative models of demographic history between *Pd*-1 and *Pd*-2. The model selection procedure led to the selection of the strict-isolation model with posterior probability of 0.77 and 0.75 with the random forest method for reference Gd293 and Gd45 respectively. The rejection method also led to the selection of the strict-isolation model in 79% and 76% of cases for reference Gd293 and Gd45 respectively. Therefore, both model selection methods support the model without contemporaneous migration. The parameters estimated from the posterior distributions of the strict-isolation model using the neural network method are presented below. Founders1 and Founders2 are the ages of the change in population size 1 and 2 respectively. Tsplit refers to the calculated divergence time measured in generations. Tdem1 and Tdem2 in the past for population size 1 and 2 respectively. The negative parameter values estimated by the neural network method have been converted to 0. 2.5%, 50% and 97.5% are the percentiles of the posterior distributions.

References	Parameters	2.5%	50%	97.5%
Gd293 (clade *Pd*-1)	N1	345,815	4,552,162	9,652,703
	N2	270,581	4,610,804	9,694,012
	Na	0	2,084,196	5,390,405
	founders1	0	789,036	3,197,127
	founders2	0	876,616	3,165,903
	Tsplit	114,669	746,675	1,537,194
	Tdem1	36,173	278,464	1,062,168
	Tdem2	568	299,977	1,077,330
Gd45 (clade *Pd*-2)	N1	231,643	4,421,257	9,573,277
	N2	217,313	4,642,649	9,678,721
	Na	285,375	1,831,036	5,543,214
	founders1	0	1,547,895	4,001,733
	founders2	0	702,227	3,041,035
	Tsplit	124,707	773,607	1,595,541
	Tdem1	22,596	272,268	1,155,328
	Tdem2	4,699	258,879	1,116,295

Table S17.

Summaries of the synteny analysis carried out with different parameters. A total of 25 analyses were carried out with different 'anchors' (minimum required number of genes [anchors] to call a syntenic block) and 'gaps' (number of upstream and downstream genes to search for anchors) parameters (see section 'Synteny' in the Methods). In all 25 microsynteny-based phylogenetic analyses, *Pd*-1 and *Pd*-2 clades were recovered as monophyletic with 100% of replicates of the bootstrap and SH-like approximate likelihood ratio test. Nnodes: number of nodes (i.e. genes) in the network; Pnodes: percentage of genes included in the network in relation to the total number of genes (sum of genes over all genomes) present in the dataset; Nedges: number of edges (links between nodes) present in the network; Nclus: number of cluster (of genes) present in the network; NclusU: number of cluster (of genes) present in the network and unique to either *Pd*-1 or *Pd*-2; PclusU: percentage of clusters that are unique to either *Pd*-1 or *Pd*-2; coef: clustering coefficient measuring the extent to which nodes in a network tend to form clusters or tightly interconnected groups; degree: the node degree is the number of edges connected to that node (i.e. how many connections or neighbours a node has); scu1/scu2: number of single-copy genes unique to *Pd*-1 and *Pd*-2 respectively; mcu1/mcu2: number of multi-copy genes unique to *Pd*-1 and *Pd*-2 respectively. Mean, median, minimum and maximum values observed across the 25 runs are also presented.

Run	anchors	gaps	Nnodes	Pnodes	Nedges	Nclus	NclusU	PclusU	coef	degree	scu1	scu2	mcu1	mcu2
1	10	15	81104	94.1	338926	9044	991	11	0.95	8.4	433	512	27	19
2	15	15	80236	93.1	327676	9173	1259	13.7	0.95	8.2	600	607	31	21
3	20	15	79256	92	317936	9271	1487	16	0.95	8	706	725	33	23
4	25	15	78490	91.1	312442	9268	1586	17.1	0.95	8	734	790	36	26
5	30	15	77977	90.5	307175	9337	1697	18.2	0.95	7.9	801	833	38	25
6	10	20	81344	94.4	340702	8939	942	10.5	0.95	8.4	404	489	31	18
7	15	20	80446	93.3	329419	9095	1179	13	0.94	8.2	547	566	48	18
8	20	20	79436	92.2	319362	9213	1400	15.2	0.95	8	670	673	36	21
9	25	20	78711	91.3	313961	9239	1524	16.5	0.95	8	717	747	36	24
10	30	20	78236	90.8	308883	9330	1662	17.8	0.95	7.9	796	806	35	25
11	10	25	81507	94.6	342063	8865	896	10.1	0.95	8.4	389	462	26	19
12	15	25	80607	93.5	330423	9037	1138	12.6	0.94	8.2	520	545	54	19
13	20	25	79689	92.5	320482	9202	1365	14.8	0.95	8	644	645	55	21
14	25	25	78919	91.6	315181	9202	1466	15.9	0.95	8	693	690	62	21
15	30	25	78441	91	310070	9312	1586	17	0.95	7.9	767	746	51	22
16	10	30	81567	94.6	342819	8835	887	10	0.94	8.4	385	458	25	19
17	15	30	80783	93.7	331310	9011	1103	12.2	0.94	8.2	515	532	37	19
18	20	30	79806	92.6	321371	9175	1355	14.8	0.95	8.1	644	632	57	22
19	25	30	79073	91.8	316065	9199	1484	16.1	0.95	8	695	696	71	22
20	30	30	78608	91.2	311118	9314	1608	17.3	0.95	7.9	773	745	69	21
21	10	35	81588	94.7	343395	8811	873	9.9	0.94	8.4	380	454	20	19
22	15	35	80800	93.8	331821	8979	1077	12	0.94	8.2	502	525	32	18
23	20	35	79854	92.7	321893	9138	1327	14.5	0.94	8.1	629	625	52	21
24	25	35	79173	91.9	316763	9157	1440	15.7	0.94	8	670	674	75	21
25	30	35	78728	91.4	311976	9266	1558	16.8	0.95	7.9	731	730	76	21
Mean	20	25	79775	92.6	323329	9136	1316	14.4	0.95	8.1	614	636	45	21
Median	20	25	79689	92.5	320482	9175	1365	14.8	0.95	8	644	645	37	21
Min	10	15	77977	90.5	307175	8811	873	9.9	0.94	7.9	380	454	20	18
Max	30	35	81588	94.7	343395	9337	1697	18.2	0.95	8.4	801	833	76	26

Table S18.

Geographic coordinates of origin estimated from the continuous assignment (SPASIBA analysis) of the 33 North American *Pd* isolates to a grid covering Europe (Figure 4). The region with inferred locations (displayed as yellow points in Figure 4) represents the most likely source population of the North American introduction of *Pd*-1.

Isolate	Latitude	Longitude
Gd_01053-	48.81387	26.68005
Gd_01111-aaa	48.81387	26.68005
Gd_01111-aab	48.81387	26.68005
Gd_01111-aba	48.81387	26.68005
Gd_01111-abb	48.81387	26.68005
Gd_01112-aaa	48.81387	26.68005
Gd_01112-aab	48.81387	26.68005
Gd_01115-aaa	48.81387	26.68005
Gd_01115-aab	48.81387	26.68005
Gd_01116-aaa	48.81387	26.68005
Gd_01116-aab	48.81387	26.68005
Gd_01117-aaa	48.81387	26.68005
Gd_01117-aab	48.81387	26.68005
Gd_01118-aaa	48.81387	26.68005
Gd_01118-aab	48.81387	26.68005
Gd_01119-aaa	48.81387	26.68005
Gd_01119-aab	48.81387	26.68005
Gd_01120-aaa	48.81387	26.68005
Gd_01120-aab	48.81387	26.68005
Gd_01121-aaa	48.81387	26.68005
Gd_01121-aab	48.81387	26.68005
Gd_01122-aab	48.81387	26.68005
Gd_01123-aaa	48.81387	26.68005
Gd_01123-aab	48.81387	26.68005
Gd_01124-aaa	48.81387	26.68005
Gd_01124-aab	48.81387	26.68005
Gd_01125-aaa	48.81387	26.68005
Gd_01126-aaa	48.81387	26.68005
Gd_01126-aab	48.81387	26.68005
Gd_01127-aaa	48.64327	26.68005
Gd_01127-aab	48.81387	26.68005
Gd_01128-aaa	48.81387	26.68005
Gd_01128-aba	48.81387	26.68005

Supplementary References

1 Lorch, J. M. *et al.* Distribution and environmental persistence of the causative agent of white-nose syndrome, *Geomyces destructans*, in bat hibernacula of the eastern United States. *Applied and Environmental Microbiology* **79**, 1293-1301 (2013).

2 Puechmaille, S. J. *et al.* Pan-European distribution of white-nose syndrome fungus (*Geomyces destructans*) not associated with mass mortality. *PLoS ONE* **6**, e19167 (2011).

3 Fischer, N. M. *et al.* Population genetics as a tool to elucidate pathogen reservoirs: Lessons from *Pseudogymnoascus destructans*, the causative agent of White-Nose disease in bats. *Mol Ecol* **31**, 675-690 (2022).

4 Palmer, J. M., Drees, K. P., Foster, J. T. & Lindner, D. L. Extreme sensitivity to ultraviolet light in the fungal pathogen causing white-nose syndrome of bats. *Nature Communications* **9**, 35 (2018).

5 Zhelyazkova, V. L., Hubancheva, A., Radoslavov, G., Toshkova, N. L. & Puechmaille, S. J. Did you wash your caving suit? Cavers' role in the potential spread of *Pseudogymnoascus destructans*, the causative agent of white-nose disease. *International Journal of Speleology* **49**, 7 (2020).

6 Scheltens, J. Cowboys, cavers and cossacks. The first NSS field trip to the Soviet Union. *NSS News* **July 1991**, 198-204 (1991).

7 LaRock, E. & Boston, P. Cave Science News: Kim Cunningham. *Journal of Cave and Karst Studies* **63**, 53-54 (2001).

8 Nicola, C. August 27 to September 15, 2005—The twelfth annual Ukraine expedition of the Ukrainian American Youth Caver Exchange Foundation (UAYCEF), an NSS Project. *NSS News* **March 2005**, 2 (2005).

9 Taylor, P. & Nicola, C. *The secret of priest's grotto*. (Kar-Ben Publishing, 2007).

10 Steele, B. An Interview with Chris Nicola (and the Priest Cave story). *NSS News* **January 2005**, 6-12 (2005).

11 Yabolkova, N. *et al*. Tributes to Alexander Klimchouk. *NSS News***August 2023**, 18-20 (2023).

12 Veilleux, J. P. Current status of white-nose syndrome in the northeastern United States. *Bat Research News* **49**, 15-17 (2008).

13 Lorch, J. M. *et al.* First Detection of Bat White-Nose Syndrome in Western North America. *mSphere* **1**, e00148-00116 (2016).

14 Salleh, S., Cox-Witton, K., Salleh, Y. & Hufschmid, J. Caver knowledge and biosecurity attitudes towards white-nose syndrome and implications for global spread. *EcoHealth* **17**, 487-497 (2020).

15 Nei, M., Tajima, F. & Tateno, Y. Accuracy of estimated phylogenetic trees from molecular data. *Journal of Molecular Evolution* **19**, 153-170 (1983).

16 IUCN. *The IUCN red list of threatened species. 2022-2.* , <https://www.iucnredlist.org> (2023).

17 Razgour, O., Ibáñez, C., Puechmaille, S. J. & Juste, J. in *Handbook of the Mammals of Europe* (eds Klaus Hackländer & Frank E. Zachos) 1-41 (Springer International Publishing, 2020).

18 Zukal, J. *et al.* White-nose syndrome without borders: *Pseudogymnoascus destructans* infection tolerated in Europe and Palearctic Asia but not in North America. *Scientific Reports* **6**, 19829 (2016).

19 Sharma, L. *et al.* Worldwide recombination in emergent white-nose syndrome pathogen *Pseudogymnoascus destructans*. *bioRxiv*, 868331 (2019).

20 Kim, Y. S., Yang, M. S., Jeong, C. G., Chung, C. U. & Oem, J. K. First Isolation of *Pseudogymnoascus destructans*, the fungal causative agent of White-Nose Syndrome, in Korean bats (*Myotis petax*). *J Fungi* (Basel) 8 (2022).
