# Supplementary material for: Two distinct host-specialized fungal species cause white-nose disease in bats
Source: Nature. 2025 May 28;642(8069):1034–40. doi: 10.1038/s41586-025-09060-5 (PMC12222008; doi:10.1038/s41586-025-09060-5)
Supplement: Supplementary file 2 — Reporting Summary [file 41586_2025_9060_MOESM2_ESM.pdf]

nature portfolio

Corresponding author(s): S.J. Puechmaille

Last updated by author(s): Mar 29, 2025

# Reporting Summary

Nature Portfolio wishes to improve the reproducibility of the work that we publish. This form provides structure for consistency and transparency in reporting. For further information on Nature Portfolio policies, see our Editorial Policies and the Editorial Policy Checklist.

## Statistics

For all statistical analyses, confirm that the following items are present in the figure legend, table legend, main text, or Methods section.

| n/a | Confirmed | |
|---|---|---|
| ☐ | ☒ | The exact sample size (*n*) for each experimental group/condition, given as a discrete number and unit of measurement |
| ☐ | ☒ | A statement on whether measurements were taken from distinct samples or whether the same sample was measured repeatedly |
| ☐ | ☒ | The statistical test(s) used AND whether they are one- or two-sided<br>*Only common tests should be described solely by name; describe more complex techniques in the Methods section.* |
| ☐ | ☒ | A description of all covariates tested |
| ☐ | ☒ | A description of any assumptions or corrections, such as tests of normality and adjustment for multiple comparisons |
| ☐ | ☒ | A full description of the statistical parameters including central tendency (e.g. means) or other basic estimates (e.g. regression coefficient) AND variation (e.g. standard deviation) or associated estimates of uncertainty (e.g. confidence intervals) |
| ☐ | ☒ | For null hypothesis testing, the test statistic (e.g. *F*, *t*, *r*) with confidence intervals, effect sizes, degrees of freedom and *P* value noted<br>*Give P values as exact values whenever suitable.* |
| ☐ | ☒ | For Bayesian analysis, information on the choice of priors and Markov chain Monte Carlo settings |
| ☐ | ☒ | For hierarchical and complex designs, identification of the appropriate level for tests and full reporting of outcomes |
| ☒ | ☐ | Estimates of effect sizes (e.g. Cohen's *d*, Pearson's *r*), indicating how they were calculated |

*Our web collection on statistics for biologists contains articles on many of the points above.*

## Software and code

Policy information about availability of computer code

| | |
|---|---|
| Data collection | No software was used. |
| Data analysis | The following software was used in analyses (full description of analyses provided in supplemental information):<br>Augustus (v3.4.0), Bedops (v2.4.41), BEDTools (v2.30.0-8), BLASTN (v2.9.0+), BUSCO (v5.2.2), bwa-mem (v.0.7.17-r1188), bwa-mem2 (v2.2.1), CD-HIT-est (v4.8.1), DIAMOND (v2.1.7), DILS (unversioned, https://github.com/popgenomics/DILS_web), EEMS (unversioned), EMBOSS (v6.6.0.0), Fastp (v0.23.4), FGT.pl script (unversioned, https://github.com/dbsloan/fgt), FigTree (v1.4.4), Flye (v2.9), funannotate pipeline (v1.8.1), gatk (v4.2.6.1), GeneMapper® (v5), Guppy (v5.0.7), hmmsearch (v3.1), HyPo (v1.0.3), Inkscape (v1.1.1), IQTREE2 (v2.0.6), LDhelmet (v1.10), MAFFT (v7.453), metaeuk (v5.34c21f2), msnsam (october 2007 version), NanoPlot (v1.42.0), NanoStat (unversioned), NUCmer4 (v4.0.0), orthoDB (v10), Picard (v2.27.1), pixy (v1.2.7.beta1), Porechop (unversioned, https://github.com/rrwick/Porechop), RepeatMasker (v.4.1.2), RepeatModeler (v2.0.1), RNA STAR mapping tool (v2.7.10b), Samtools (v1.16.1), SeqKit (v0.16.1), SNAPE-pooled (unversioned, https://github.com/EmanueleRaineri/snape-pooled), Splitstree CE (v6.3.27), vcftools (version modified by J. Dutheil; https://github.com/jydu/vcftools) as well as R software (v4.1.1) with the packages abc (v2.2.1), abcrf (v1.9), adegenet (v2.1.5), adehabitatHR (v0.4.21), adimpro (v0.9.6), ape (v5.7.1), brms (v2.20.3), EBImage (v4.3), ggmap (v3.0.2), ggplot2 (v3.5.0), hierfstat (v0.5.11), INLA (v0.0.4), ks (v1.14.1), MonoPhy (v1.3.2), performance (v0.12.4), Phangorn (v2.11.1), poolfstat (v2.0.0.), poppr (v2.9.3), sp (v1.4-6), SPASIBA (v24.6.27), Syntenet (v1.8.1), reemsplots2 (v0.1.0), rworldxtra( (v1.01) and rworldmap (v1.3.8). |

For manuscripts utilizing custom algorithms or software that are central to the research but not yet described in published literature, software must be made available to editors and reviewers. We strongly encourage code deposition in a community repository (e.g. GitHub). See the Nature Portfolio guidelines for submitting code & software for further information.

# Data

Policy information about availability of data

All manuscripts must include a data availability statement. This statement should provide the following information, where applicable:

- Accession codes, unique identifiers, or web links for publicly available datasets
- A description of any restrictions on data availability
- For clinical datasets or third party data, please ensure that the statement adheres to our policy

The genomic sequences and assembled genomes have been deposited under accession numbers SRR30476767–SRR30476787 and SRR30476795–SRR30476796 (see Table S10), while the PoolSeq data are available under accession numbers SRR30476788–SRR30476794 and SRR30476766 (see Table S15). These data can be accessed via BioProject PRJNA862744 at the National Center for Biotechnology Information. Metadata along with microsatellite genotypes data are provided in Table S1 (Exact site locations are not disclosed to protect endangered species and landowners), temperature, absolute humidity and M. daubentonii presence in Table S3, colouration of agar medium in Table S8, and colony expansion rates in Table S9. All other data are available in the manuscript or the supplementary information.

# Research involving human participants, their data, or biological material

Policy information about studies with human participants or human data. See also policy information about sex, gender (identity/presentation), and sexual orientation and race, ethnicity and racism.

| | |
|---|---|
| Reporting on sex and gender | N/A |
| Reporting on race, ethnicity, or other socially relevant groupings | N/A |
| Population characteristics | N/A |
| Recruitment | N/A |
| Ethics oversight | N/A |

Note that full information on the approval of the study protocol must also be provided in the manuscript.

# Field-specific reporting

Please select the one below that is the best fit for your research. If you are not sure, read the appropriate sections before making your selection.

☐ Life sciences ☐ Behavioural & social sciences ☒ Ecological, evolutionary & environmental sciences

For a reference copy of the document with all sections, see nature.com/documents/nr-reporting-summary-flat.pdf

# Ecological, evolutionary & environmental sciences study design

All studies must disclose on these points even when the disclosure is negative.

| | |
|---|---|
| Study description | Characterisation of ecological and genetic differentiation across Eurasian and North American samples of the fungus Pseudogymnoascus destructans |
| Research sample | Reference collection of 5,479 isolates of Pseudogymnoascus destructans sampled primarily across Eurasia (33 isolates from North America). In addition, 11 Pseudogymnoascus destructans isolates (10 from Eurasia and 1 from North America) were selected for full genome long-read sequencing and were analysed together with 7 previously published Pseudogymnoascus destructans Genomes (5 from Europe, 1 from Mongolia, 1 from China; Drees et al., 2017). Furthermore, a total of 132 isolates from this study were used for pooled Illumina sequencing (split into two pools). |
| Sampling strategy | We aimed to sample as many sites as feasible throughout Europe while keeping a good geographical representation. In total, we obtaind 5,446 samples from 256 sites in 26 countries in Europe, offering an important geographic coverage of the suspected region of origin. For North America, given the clonal origin of the introduction, a much lower sample size (N=9 sites and 33 isolates) was needed. |
| Data collection | Our work adhered to the ethical wildlife research guidelines of the American Society of Mammalogists for the use of wild mammals in research and education (Sikes et al. 2016).<br>Swab samples of Pseudogymnoascus destructans were collected from within bat hibernacula. Sampling from hibernating bats was conducted without capture or handling, by collecting samples while the bats remained freely hanging. The samples were collected by lightly swabbing the infected areas with a sterile dry swab. This method is considered as minimally invasive or even non-invasive. This work was completed with the help from A. Bezard, A. Kubátová, Aleksandra Lange, Alessandra Peron, Alex Lefevre, Alexander Lazarov, Alexandra Telea, Alexandre Cartier, Alina Larion, Alphonse Malpel, Amanda Davies, Andres Beck, Andrew Brinckman, Andriy |

Melnychuk, Andrzej Kepel, Andrzej Wojtaszewski, Angel Ivanov, Àngel Torrent, Ann Lenaerts, Anna Roswag, Anna Suvorova, Anne-Jilke Haarsma, Anne Petzold, Annika Breitfelder, Anthony Lane, Anthony Le Nozahic, Anthony Nickson, Antonia Hubancheva, Artem Tarasov, Ash Murray, Atanas Stavrev, Axel Donning, Axel Griesau, Axel Keusemann, Bart Mulkens, Benjamin Meme-Lafond, Bernd Ohlendorf, Bernhard Walk, Beytullah Özkan, Blanka Lehotská, Boris Petrov, Brian Briggs, Brigitte Meiswinkel, Carlos Ibáñez, Carsten Dense, Catherine Reilly, Chris Vine, Christian Dietz, Christian Jungmann, Christian Sebening, Christoph Treß, Christophe Borel, Christophe Parisot, Christopher Paton, Claudi Gebhart, Clemens Kliesch, Colin Morris, Corentin Le Floch, Csaba Jére, Damian Celiński, Dana Wagemakers, Daniel Eva, Daniela Hamidovic, Daniela Pilgrim, Daniela Schmieder, Daniela Wieser, Dave Hughes, David Anderson, David Aupermann, David Dodds, David Endacott, David García Jiménez, David Hellmann, David Patterson, David Wills, Didier Montfort, Dieter Hülshoff, Dieter Sulzbacher, Dimitar Kunev, Dirk Karoske, Dragoș Bălășoiu, Ebbe Nytors, Eeva-Maria Kyheröinen, Egoitz Salsamendi, Elena Migens Maqueda, Emrah Çoraman, Erich Taube, Ernst Auer, Eva Kriner, Ewa Przepiorka, Fabio Bontadina, Fabio Suppini, Fiona Parker, Florian Gloza-Rausch, Francesco Grazioli, Frank Meisel, Frauke Meier, Frédéric Forget, Frédéric Touzalin, Fulgencio Lison, Gabriella Krivek, Gaël Verat, Gary Shears, Georg Warnke, Gerald Kerth, Gerald Larcher, Giazarian, Goran Rnjak, Gregory Beneux, Grzegorz Apoznański, Grzegorz Lesinski, Guilia Console, Gunars Petersons, Gunther Capo, Gustav Dinger, Gwenaelle Hurpy, Gwendoline Dumenil, H. Bandouchova, H. Seimers, Hannes Köble, Harald Mixanig, Heino Hauf, Helen Miller, Henryk Hörner, Holger Schütt, Hubert Baltus, Iain Hysom, Ian Bond, Ilaria Vaccarelli, Ilona Imoberdorf, Ilze Brila, Inazio Garin, Ingrid Heißen, Ingrid Oftedal, Irbin Manuel Veliz Isidro, Ireneusz Ruczynski, Irina Pocora, Irina Würtele, István Csősz, Ivailo Borissov, Ivan Napotnik, Ivana Budinski, J. Flousek, J. Nogueras, J. Pikula, J. Zukal, J.L. Gathoye, Jamie Shadbolt, Jan Boshmer, Jane Harris, Jane Sedgeley-Strachan, Jasmin Pašić, Jasminko Mulaomerović, Jean-Yves Courtois, Jean Guhring, Jenny Harris, Jens Berg, Jens Krüger, Jens Rydell, Jeroen van der Kooij, Joachim Frömert, John Haddow, Johnny de Jong, Jörn Horn, Jose Siles, Juan R. Boyero, Julia Prüger, Juliane Schatz, Jurgis Suba, Justyna Błesznowska, Karina Jungmann, Karsten Passior, Katharina Bürger, Kathy Warden, Kees Mostert, Kerstin Genz, Klaus Heck, Kristof De Clercq, Krum Sirakov, Krzysztof Piksa, Kseniia Kravchenko, Laura Torrent, Laurence Florian, Laurent Arthur, Lauri Lutsar, Lea Bütje, Lena Godlevska, Lena Grosche, Lide Jimenez, Lilian Girard, Lionel L'Hoste, Lisa Worledge, Llorenc Capella Ripoll, Loic Robert, Lotte Gielis, Lucretia Deplazes, Ludovic Jouve, Luis Vicente, Luisa Rodrigues, Lyn Wells, M. Kubešová, M. Orlova, Magda Milczarska, Maik Korreng, Manfred Keller, Manuel Graf, Manuela Schult, Mara Calvini, Marcin Rusinski, Maria Das Neves Paiva Cardoso, Marion Laprun, Markus Melber, Markus Milchram, Markus Schmidberger, Markus Thies, Martin Biedermann, Martin Harder, Martin Koch, Martin Starrach, Martina Palmer, Mathijs Borms, Matija Perne, Matthias Göttsche, Matthias Hammer, Matthias Weiß, Matthias Zizelmann, Mauro Mucedda, Mechthild Höller, Michael Frede, Miguel Àngel Fuentes Rosua, Mike Debret, Mirna Mazija, Momchil Naydenov, Monika Podgorelec, Morten Elmeros, N. Martinkova, Nataša Sivec, Nia Toshkova, Nick Tribe, Nicolas Cayssiols, Nicolas Fasel, Nicola Fischer, Niklois Jungbluth, Nina Hagner-Wahlsten, Norbert Röse, Nuno Pinto, O. Orlov, Oliver Kalda, Oleksandr Vikyrchak, Olvido Tejedor, Oscar de Paz, P. Blažková, P. Schnitzerová, P. Táje, P. Tájek, Paola Culasso, Pascal Bellion, Pascal Giosa, Pascal Verdeyroux, Patty Briggs, Paul Hope, Paweł Kmiecik, Per IngeVærnesbranden, Peter Busse, Peter Heubes, Peter Holtz, Peter Smith, Petra Gatz, Petra Žvorc, Petro Ploshchanskyi, Philippe Defernez, Philippe Theou, Pierre-Emmanuel Bastien, Piotr Zielinski, Primož Presetnik, Quentin Smits, Radek Lučan, Radostina Tsoneva, Rainer Marcek, Ralf Hansen, Ralf Koch, Rasit Bilgin, Rauno Kalda, Reimund Francke, Reinhard Koch, Rémi Hanotel, Rich Flight, Roberto Toffoli, Robin Moffitt, Ruddy Cors, S. Rebrov, Sabine Lind, Sabine Portig, Sam Dyer, Sándor Boldogh, Sandra Möller, Sebastian Petters, Sebastien Puechmaille, Serbülent Pakzuz, Serena Dool, Serena Magagnoli, Sheelagh Kerry, Shirley Thompson, Simone Pysarczuk, Stamen Dimitrov, Stanimira Deleva, Stefan Schürmann, Steffi Pfeiffer, Stephanie Wohlfahrt, Steve Parker, Stoyan Goranov, Sue Lane, Susan Kerwin, Susanne Rosenau, Szilárd Bücs, T. Juhnke, Tamás Görföl, Tarik Dervović, Tea Knapič, Teodor Jhotev, Thomas Bormann, Thomas Cheyrezy, Thomas Frank, Thomas Kuß, Thomas Le Campion, Thomas Lilley, Tiago Brito, Tina Aughney, Tino Staudt, Todor Karakiev, Tom Hastings, Tom McOwat, Tomasz Kokurewicz, Toni Watt, Tony Lane, Torsten Blohm, Tsvetan Ostromsky, Ulrich Zöphel, V. Kovacova, V. Lensinger, V.S. Crukov, Vesselin Zhelyazkov, Victor Senderov, Victoria Nistreanu, Viktor Ilyukha, Viorel Pocora, Vita Hommersen, Vitaliy Guckov, Vivien Sottejean, Vladislav Caldari, Vlashenko, Volker Kubisch, Weigert Steen, Wigbert Schorcht, Winfried Krämer, Wolfgang Fiedler, Wolfgang Otremba, Wolfgang Rackow, Wolfgang Strittmatter, Xavier Mestdagh, Yana Dimova, Yann Gager, Yann Le Bris, Yannick Beucher, Yvon Guenescheau, Zuzanna Halat.

| | |
|---|---|
| Timing and spatial scale | The samples were collected between 02.02.2008 and 11.03.2022 based on feasability and availability of bats. The timing of the sampling was of no interest to the study design and adressed research questions. |
| Data exclusions | No data were excluded from the analyses. |
| Reproducibility | In Table S1, we provide a genotype table of all isolates and necessary additional information (metadata) for a full reproducibility of all the analyses. Similarly, we provide GenBank accession numbers of raw sequences and genomes produced in this study. All analyses are described in detail in the Material and Methods section to allow full reproducibility. |
| Randomization | There was no randomisation in the study considering it was an observational study of different individuals and their genotypes and genomes. |
| Blinding | There was no blinding in the study as this was a descriptive study design not an experimental one. |

Did the study involve field work? ☒ Yes ☐ No

## Field work, collection and transport

| | |
|---|---|
| Field conditions | Field work was conducted for sample collections from temperate hibernacula, ususally in the winter season. A total of 264 sites were sampled with conditions varying between different sites and years though cool and humid conditions were typical. |
| Location | 264 sites were sampled across Eurasia and North America (see Table S1). Exact site locations are not disclosed to protect endangered species and landowners. |
| Access & import/export | This work was conducted under permission from the following authorities:<br>Italy: Regional Speleological Federation of Emilia-Romagna (F.S.R.E.R.), and the Management Bodies of the Parks of Emilia-Romagna;<br>Poland: Genarny Dyrektor Ochrony Środowiska (General Director for Environmental Protection); Regional Directorate for |

Environmental Protection in Gorzów Wielkopolski (Regionalna Dyrekcja Ochrony Środowiska w Gorzowie Wielkopolskim); Switzerland: Kantonaler Fledermausschutz Aargau; Germany: Umweltamt, Veterinäramt; Untere Landschaftsbehörde Siegen-Wittgenstein; Untere Naturschutzbehörde Umweltamt Landkreis Harz & Referat Verbraucherschutz, Veterinärangelegenheiten Landesverwaltungsamt Sachsen-Anhalt; Untere Naturschutzbehörde des Landkreises Vorpommern-Greifswald; Regierung von Unterfranken, Regierung von Mittelfranken; Struktur- und Genehmigungs Direktion Nord/Süd, NLWKN Niedersächsischer Landesbetrieb für Wasserwirtschft, Küsten- und Naturschutz, Region Hannover - Fachbereich Umwelt; Austria: Department of nature conservation for Carinthia, Lower Austria, Upper Austria, Salzburg, Styria and Vorarlberg; Hungary: Pest Megyei Kormányhivatal, Országos Környezetvédelmi, Természetvédelmi és Hulladékgazdálkiodási Főosztály (Pest County Government Office, National Department of Environment Protection, Nature Conservation and Waste Management); Ministry of Environment and Water; Bulgaria: Bulgarian Ministry of the Environment and Water; France: DDTM-Morbihan; DREAL; Republic of Latvia: Nature Conservation Agency; Belgium: Gouvernement Wallon; Denmark: The Nature Agency and Daugbjerg Kalkgruber; Romania: Speleological Heritage Commission; Estonia: Estonian Environmental Board; England: Natural England; Finland: Southwest Finland Centre for Economic Development, Transport and the Environment; Sweden: Uppsala djurförsöksetiska nämnd; Swedish board of Agriculture; Swedish Environmental Protection Agency; Norway: Miljødirektoratet; Luxemburg: Ministère du Développement durable et des Infrastructures du Luxembourg; Croatia: Croatian Ministry of Environment and Nature; Russian Federation: Game Management Directorate of the Republic of Karelia; Institute of Plant and Animal Ecology, Ural Division of the Russian Academy of Sciences; Slovak Republic: Ministry of the Environment of the Slovak Republic, Department of State Administration for Nature and Landscape Protection; The Netherlands: Dutch Ministry of Economic affairs; Republic of Moldova: Government of Republic of Moldova - Ministry of Environment.

| | |
|---|---|
| Disturbance | Swab samples were collected in a minimally invasive or non-invasive manner, without handling the animals. Disturbance from human presence (e.g., time spent at the site) and noise was kept to a minimum, following the procedures typically used during regular hibernacula counts. |

# Reporting for specific materials, systems and methods

We require information from authors about some types of materials, experimental systems and methods used in many studies. Here, indicate whether each material, system or method listed is relevant to your study. If you are not sure if a list item applies to your research, read the appropriate section before selecting a response.

## Materials & experimental systems

| n/a | Involved in the study |
|---|---|
| ☒ | ☐ Antibodies |
| ☒ | ☐ Eukaryotic cell lines |
| ☒ | ☐ Palaeontology and archaeology |
| ☒ | ☐ Animals and other organisms |
| ☒ | ☐ Clinical data |
| ☒ | ☐ Dual use research of concern |
| ☒ | ☐ Plants |

## Methods

| n/a | Involved in the study |
|---|---|
| ☒ | ☐ ChIP-seq |
| ☒ | ☐ Flow cytometry |
| ☒ | ☐ MRI-based neuroimaging |

## Plants

| | |
|---|---|
| Seed stocks | NA |
| Novel plant genotypes | NA |
| Authentication | NA |
