# Supplementary material for: Two distinct host-specialized fungal species cause white-nose disease in bats
Source: Nature. 2025 May 28;642(8069):1034–40. doi: 10.1038/s41586-025-09060-5 (PMC12222008; doi:10.1038/s41586-025-09060-5)
Supplement: Supplementary file 3 — This file presents a phylogenetic tree constructed from 18 microsatellites for all isolates, including clade information, bat species of origin and sampled substrates. [file 41586_2025_9060_MOESM3_ESM.pdf]

Figure S2

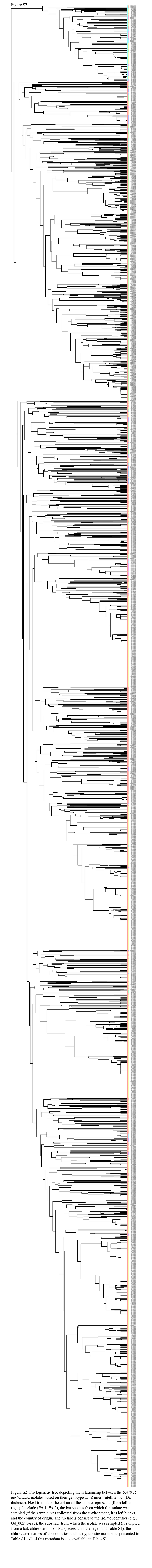

Figure S2: Phylogenetic tree depicting the relationship between the 5,479 *P. destructans* isolates based on their genotype at 18 microsatellite loci (Da distance). Next to the tip, the colour of the square represents (from left to right) the clade (*Pd*-1, *Pd*-2), the bat species from which the isolate was sampled (if the sample was collected from the environment, it is left blank), and the country of origin. The tip labels consist of the isolate identifier (e.g., Gd_00293-aad), the substrate from which the isolate was sampled (if sampled from a bat, abbreviations of bat species as in the legend of Table S1), the abbreviated names of the countries, and lastly, the site number as presented in Table S1. All of this metadata is also available in Table S1.
